# Neural mechanisms of negative emotionality and cognitive control: The role of frontal midline theta

**DOI:** 10.3758/s13415-026-01434-0

**Published:** 2026-04-14

**Authors:** Collin D. Teich, Eric Rawls, Kara L. Stevens, Steven S. S. Kang, Nicholas D. Davenport, Scott R. Sponheim, Melissa A. Polusny, Craig A. Marquardt

**Affiliations:** 1 Department of Psychiatry and Behavioral Sciences, University of Minnesota, Minneapolis, MN USA; 2 Minneapolis Veterans Affairs Health Care System, Minneapolis, MN USA; 3 School of Medicine, University of Missouri-Kansas City, Kansas City, MO USA; 4 Department of Psychology, University of Minnesota-Twin Cities, Minneapolis, MN USA; 5 Center for Care Delivery and Outcomes Research, Minneapolis, MN USA; 6 Minneapolis VA Medical Center, One Veterans Drive (B68-2), Minneapolis, MN 55417 USA

**Keywords:** Cognitive control, Decision-making, Frontal midline theta, Personality, Electroencephalography, Military

## Abstract

Early adulthood is characterized by the emergence of many forms of psychopathology as well as the development of cognitive abilities necessary for adaptive functioning (e.g., cognitive control). This study investigated brain responses that mediate the relationship between the ability to inhibit unwanted behavior—a core function of cognitive control—and personality characteristics associated with the development of psychopathology. Using a Go/No-Go task, we examined how theta and alpha frequency activity (3–8 and 10–20 Hz, respectively) within electroencephalography (EEG) recorded over middle frontal regions of the scalp (i.e., frontal midline theta [FMT]) may be sensitive to the effects of personality traits of Emotionality, Constraint, and Absorption, traits with well-studied connections to psychopathology and psychopathology risk factors, on behavioral responses reflecting cognitive control. Young adult United States Army National Guard recruits (n = 106) completed a Go/No-Go task that varied in difficulty across three blocks to magnify cognitive demands. FMT (electrode FCz) and behavioral performance varied as a function of the task demands, and FMT was associated with negative emotionality (NEM) and absorption. Desynchronization of alpha frequencies was evident at occipital electrodes (PO7, PO8) and related to task block difficulty. A mediation analysis revealed that FMT explained (mediated) the association between heightened NEM and worse performance on the Go/No-Go task. No such effects were evident for alpha desynchronization. Findings are consistent with FMT during control of behavioral responses being a mechanism by which a propensity toward experiencing negative emotions such as anxiety, fear, and stress (i.e., NEM) compromises cognitive control and self-regulation.

**Supplementary Information:**

The online version contains supplementary material available at 10.3758/s13415-026-01434-0.

## Introduction

Cognitive control encompasses an ensemble of closely connected mental processes including attention (Mackie et al., 2013; Posner et al., 1975), working memory (Engle & Kane, 2004; Redick, 2014; Unsworth et al., 2012), inhibitory control (Cohen et al., 1990; Goghari & MacDonald, 2009), and conflict monitoring (Botvinick et al., 2001; Yeung et al., 2013). These mental processes are used in concert: attention selectively filters information and allocates cognitive resources, working memory facilitates the temporary storage and manipulation of information, inhibitory control suppresses irrelevant responses, and conflict monitoring detects deviation from environmental and behavioral expectations. Together, they form an integrated system essential for goal-directed behavior (Badre & Wagner, 2004). Cognitive control is also deeply intertwined with emotion (Ochsner & Gross, 2005), because it facilitates adaptive responding to environmental challenges (Hendricks & Buchanan, 2016; Saunders et al., 2015). For example, cognitive control may be employed to redirect attention away from threatening stimuli, reappraise the meaning of distressing situations, or inhibit impulsive emotional reactions (Angelidis et al., 2018; Euler et al., 2014; Peckham & Johnson, 2018). Furthermore, emotion can disrupt cognitive control by changing the allocation of attention away from the task at hand (Eysenck et al., 2007). The interaction of emotion with cognitive control is manifest via neurophysiological activations of frontal cortices, especially midline anterior cingulate and dorsolateral prefrontal regions (Etkin et al., 2015; Goldin et al., 2008). In the present study, we sought to examine the effect of affective traits on neural functions of cognitive control. In a sample of National Guard recruits preparing to ship to basic combat training (BCT), we administered a challenging laboratory-based Go/No-Go task while acquiring electroencephalography (EEG). We specifically investigated how individual differences in enduring affective traits might alter frontal neural functions tied to cognitive control to deepen our understanding of how such traits in early adulthood might confer risk for psychopathology after stressors, such as military training.

Frontal midline theta-band (FMT) activity, defined as frontocentral (e.g., FCz electrode) brain activity between approximately 3 to 8 Hz, is a promising neural marker of cognitive control (Buzzell et al., 2019; Cavanagh & Frank, 2014; Cavanagh & Shackman, 2015; Eisma et al., 2021; Nigbur et al., 2012; van Noordt et al., 2022). Frontal midline theta-band is modulated by a variety of task conditions designed to manipulate attention (Ishii et al., 1999), working memory load (Onton et al., 2005), and the need for inhibitory control (Messel et al., 2021; van Noordt et al., 2022). Go/No-Go tasks are often used to evoke these neural processes with and without the use of emotional task manipulations. Participants are typically presented with a series of targets requiring rapid responses, but targets are intermixed with non-target stimuli. Successful disengagement from nontargets requires attentional vigilance and the inhibition of prepotent responses—abilities known to be connected with FMT (Dippel et al., 2016, 2017; Huster et al., 2013). For example, Lewis et al. (2006) used pairs of letters as stimuli in which participants were instructed to press a button for each letter they saw, and any repeated letter was a “No-Go” trial, which required response inhibition. They noted increased N2 amplitude, an event-related potential (ERP) component thought to be driven by phase-locked FMT (Cavanagh et al., 2009; Luu et al., 2003, 2004; Trujillo & Allen, 2007) during No-Go trials compared with Go trials. Additionally, Hong & colleagues (2020) used a spatial cueing Go/No-Go task requiring covert attention to either left or right visual fields before making go/no-go discriminations. The attended No-Go trials produced greater FMT activity than both the attended Go trials and Go/No-Go ignored trials, thereby supporting FMT as important for the deployment of cognitive effort.

In addition to theta-band activity, posterior alpha oscillations (8–14 Hz) are closely tied to cognitive control, particularly through their role in regulating sensory processing and selective attention. When alpha power is reduced, it indicates a rebalancing toward task-positive network activity that supports active cognitive processing, whereas increases in alpha power may reflect a shift toward task-negative network dominance and suppression of cognitive engagement (Foxe & Snyder, 2011; Klimesch, 2012). Go/No-Go paradigms and related inhibitory control tasks have consistently demonstrated alpha suppression over occipital regions during target processing, supporting its role as a complementary neural mechanism of attentional engagement (Mazaheri et al., 2009). Thus, examination of both theta and alpha activity provides a broader account of the neural dynamics and simultaneous neural processes supporting cognitive control.

Research has demonstrated that FMT is also related to emotional activity and its regulation. Negative emotions elicited by unexpected social rejection appear to modulate FMT (Van der Molen et al., 2017). Also, individuals with both higher emotional intelligence and emotional sensitivity show increased FMT when evaluating faces for emotional content (Knyazev et al., 2009), highlighting the importance of FMT in navigating interpersonal communication and relationships. Additionally, Ertl et al. (2013) showed that FMT activity increased when participants were asked to effortfully suppress or heighten negative emotions invoked by negatively valenced stimuli compared with a “maintain” control condition. Similarly, Zouaoui et al. (2023) found that frontal theta was increased not only in response to passive viewing of aversive compared with neutral stimuli but was also amplified when participants were asked to increase their emotional reactivity to the aversive stimuli. Because it can be advantageous to suppress the experience of negative emotions during stressful situations, these findings suggest that FMT reflects a mechanism central to this ability. Additionally, FMT has been shown to act as a salience signal (i.e., a neural indicator of behaviorally relevant or unexpected events) that is sensitive to errors (Jonker et al., 2021; Luu & Tucker, 2001; Luu et al., 2004; Trujillo & Allen, 2007).

Disruptions in cognitive control and emotion regulation processes are prevalent across various psychopathologies (Luciana & Collins, 2022; McTeague et al., 2017), yet the role of FMT activity is not uniform across psychiatric conditions. Research on FMT and psychopathology has predominantly focused on its association with anxiety. In a comprehensive meta-analysis examining the relationships between cognitive control, FMT, and anxiety, Cavanagh & Shackman (2015) concluded that FMT is maladaptively elevated in individuals with trait anxiety. In contrast, studies exploring the relationship between FMT and posttraumatic stress disorder (PTSD) have produced more varied findings. Some report increased FMT in PTSD (Cohen et al., 2013; Rawls et al., 2025), and others show attenuated FMT (DeLaRosa et al., 2020), despite using similar paradigms. This variability is particularly noteworthy given the high comorbidity between anxiety and PTSD (Bruce et al., 2001; Karr & Bastia, 2006; Newman et al., 2010; Przeworski & Dunbeck, 2016) and demonstrates how subtle differences in psychopathology measurement may influence the observed relationships with FMT.

Given evidence that FMT is relevant to several forms of internalizing psychopathology (and risk factors for those conditions), but with inconsistent findings regarding the exact nature of the association, we pursued a transdiagnostic approach in the current investigation. Traditional, group-based diagnoses of psychiatric disorders disregard clinically significant variations, and individuals that fall below diagnostic thresholds for one or more disorders are not considered (Krueger & Eaton, 2015). The categorical view also assumes each disorder is distinct from one another, when there are often multiple comorbidities that span these diagnoses (Eaton et al., 2010). Transdiagnostic factors may underlie the shared vulnerabilities across different diagnostic categories and are relevant to the development and maintenance of a broad range of psychiatric disorders (e.g., HiTOP; Kotov et al., 2017). As the field of psychopathology research has shifted to this more dimensional approach, it has become increasingly evident that transdiagnostic factors exhibit a stronger association with neurobiological foundations of behavior than traditional categories (Caspi et al., 2014; Michal et al., 2024; Patrick et al., 2013). Trait Negative Emotionality (NEM), a broad trait scale of the Multidimensional Personality Questionnaire (MPQ; Patrick et al., 2002), is a transdiagnostic predictor of psychopathology and has been associated with various forms of affective dysregulation, including anxiety, depression, and substance use disorders (Leen-Feldner et al., 2004; McGue et al., 1997; Wolf et al., 2012). Negative Emotionality reflects an individual's propensity for emotional volatility under stress-inducing conditions and their tendency for perceiving threats during social interactions. Negative Emotionality has also been associated with neural error monitoring; individuals high in NEM have smaller error-related negativity (ERN; Luu et al., 2000), a component that has been shown to be driven by theta-band oscillations (Luu et al. 2004). Other MPQ scales include trait Positive Emotionality (PEM), Constraint (CON), and Absorption. Positive Emotionality, defined as an individual’s tendency to experience and express positive emotions across broad contexts, and CON, a measure of an individual’s level of self-control over impulsive behaviors, harm avoidance, and traditionalistic values, are considered higher-order dimensions of personality along with NEM. These three dimensional traits are related to developmental models of temperament and converge with other personality frameworks (Harkness et al., 1995; Krueger, 1999; Waller et al., 1991). Meanwhile Absorption, a trait separate from PEM, NEM, and CON, is thought to be related to individual variation in openness to absorbing and engaging sensory and imaginative experiences and may reflect an individual’s proclivity to be deeply involved in their thoughts, feelings, and sensory perceptions (Tellegen & Atkinson, 1974).

Much of the research in this domain has focused specifically on the relationship between anxiety and FMT, while the association between FMT and NEM has remained largely unexplored. Given the potential significance of FMT as a neural mechanism contributing to the perturbation of negative affect that can lead to psychopathology, we sought to examine the relationship between FMT and cognitive control within the context of individual differences in MPQ traits. The relationship of NEM to FMT and cognitive control may prove important to understanding individual differences in resilience to psychological stressors (Masten et al., 1999), as NEM has been shown to be relevant in explaining PTSD within military populations (Miller, 2003). We studied a sample of 106 United States Army National Guard recruits and hypothesized that NEM would be associated with reduced executive control, as indexed by lower d-prime (d′) scores, and that this association would be explained (mediated) by low FMT. Such a finding would point to a neural process linking a propensity for emotion-related psychopathology to compromised cognitive functions and could serve as a marker of vulnerability to psychopathology or resilience, and a target for intervention.

## Methods

### Participants and procedures

Participants in this study were Minnesota National Guard recruits ages 18 and older taking part in a larger longitudinal cohort study (Polusny et al., 2023). Participants were provided with consent letters. Study team members verbally clarified the study objectives, emphasized the voluntary aspect of participation, and provided assurance of data confidentiality, even from National Guard command. All study procedures were approved by the institutional review boards of the University of Minnesota and Minneapolis Veterans Affairs Health Care System. To be eligible, participants were required to be a Minnesota Army National Guard recruit who had not participated in Basic Combat Training (BCT) and had no previous military experience (for additional details, see Polusny et al., 2023). Baseline survey data was gathered by investigators via Qualtrics in classroom settings at local armories.

A subset (N = 123) of ARMOR participants were enrolled in a sub-study in which they completed laboratory visits to measure neurobiological processes before and after BCT (see Table 1 for demographics information for the sample included in this analysis). On a rolling basis over a 2-year period, we identified participants at relatively high risk (72/123, 58.5%) versus low risk (48/123, 39%) of poor psychosocial functioning. Selection was based on a predictive algorithm developed during the pilot phase of this project, which holistically ranked participants using multiple self-report indicators of psychopathology and role functioning (for details of the full procedure of the ARMOR study and substudy, see Polusny et al., 2023). Thus, the laboratory substudy sample was enriched to capture the full range of overall functioning observed within the larger study cohort. Recruitment and assessment with EEG occurred after baseline self-report data collection (53 days on average), but before participants were deployed to BCT.

**Table 1:** Demographics

	n	%	M	SD
**Age (yr)**	106		20.2	3.62
**Gender**				
Male	71	67.0		
Female	33	31.1		
Prefer not to answer	1	0.9		
**Race**				
White	67	63.2		
Black or African American	10	9.4		
American Indian or Alaska Native	2	1.9		
Asian	8	7.5		
Other	2	1.9		
Native Hawaiian or Pacific Islander	0	0.0		
Multiracial	15	14.2		
**Ethnicity**				
Hispanic	14	13.2		
Non-Hispanic	90	84.9		
**Student status**				
Enrolled	65	61.3		
Not enrolled	40	37.7		
**Completed education (years)**				
Some high school	20	18.9		
GED	0	0.0		
High school diploma	34	32.1		
Some college	34	32.1		
Associate’s degree	9	8.5		
4-year college degree	6	5.7		
Graduate degree	2	1.8		
**Employed outside National Guard**				
Yes	82	77.4		
No	19	17.9		
Student only				

Simple demographics information for the sample. One subject had missing data for all categories, one subject had missing data for race and ethnicity, and five subjects had missing employment information

Participants completed the MPQ-155 (“Brief Form”; Patrick et al., 2002) as part of the baseline survey. Multidimensional Personality Questionnaire broad trait scales were computed from the primary trait scale scores by using proprietary weighting algorithms developed from the MPQ normative dataset. Each broad trait score is differentially driven by specific subsets of primary trait scales, with the heaviest weightings assigned as follows: Wellbeing (fun-loving, happy disposition, optimistic), Social Potency (dominance, persuasive, leadership), Achievement (ambitious, persistent), and Social Closeness (warmth, affectionate, sociable) for PEM; Stress Reaction (prone to nervousness, capriciousness), Alienation (feelings of betrayal or exploitation), and Aggression (vengeful, willingness to distress others) for NEM; Control (planful, rational), Harm Avoidance (dislikes risk), and Traditionalism (conventional standards of morality and conduct) for CON. Absorption stands alone as a primary scale and is not included in the broad trait weighting equations. There are also two validity scales in the MPQ-155 to measure noncontent-based response invalidity. The True Response Inconsistency scale (TRIN) is a set of matched questions typically endorsed in opposite directions to quantify excessive nay- or yea-saying. The Variable Response Inconsistency scale (VRIN) is another set of paired questions to assess random responding. Based on MPQ-155 normative data, participants were excluded (n = 4 excluded from the original 110 subjects with valid EEG data) with VRIN scores three standard deviations (SD), TRIN scores ± 3.21 SD, or + 2 SD VRIN and ± 2.28 SD TRIN scores away from the normative data mean.

### Task description

**Fig. 1:**
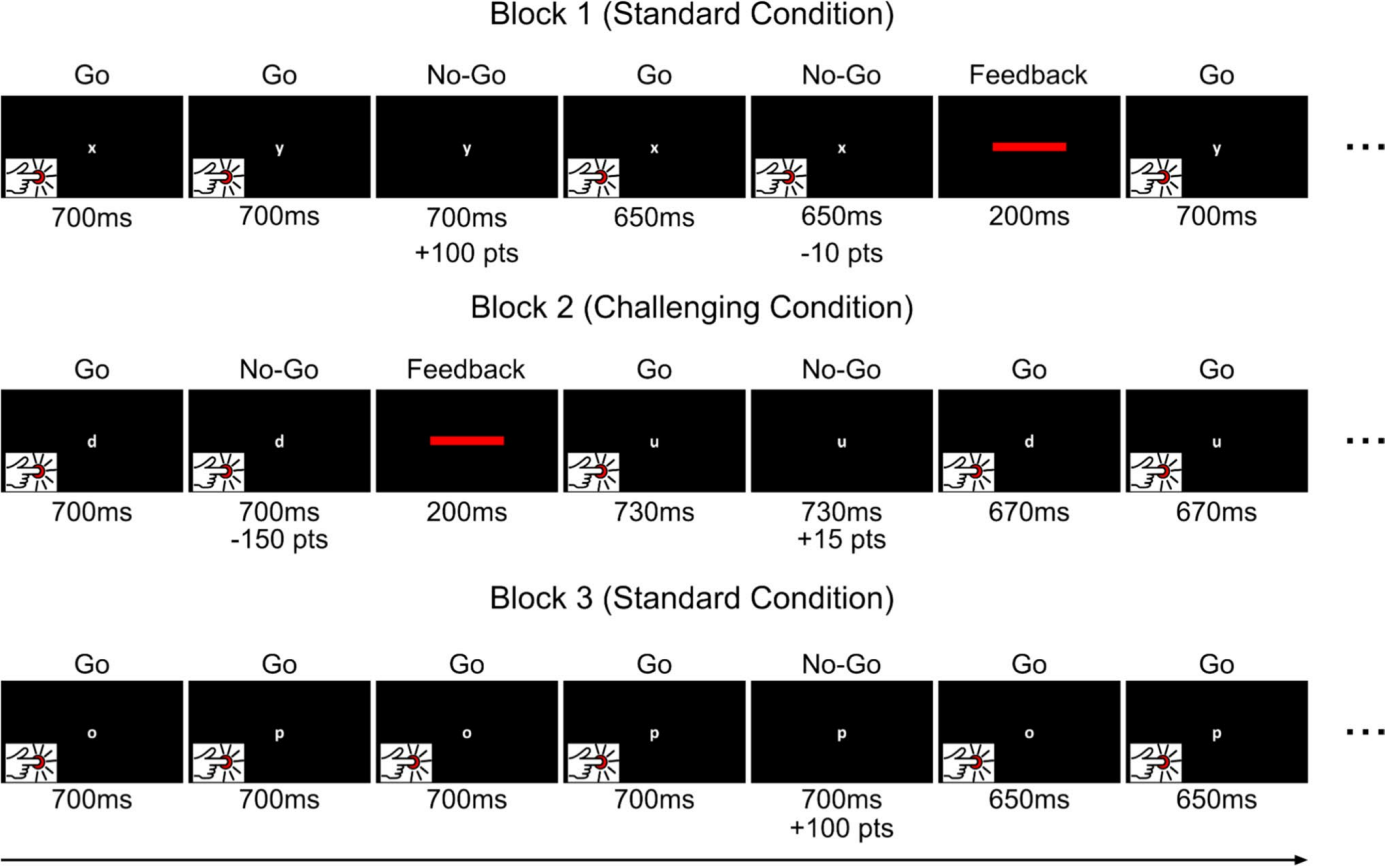
Trial by trial depiction of the Go/No-Go task employed. Each block utilized two different letters, and either letter could be a “Go” or “No-Go” trial. The times listed below each stimulus picture is indicative of both the stimulus duration and response window, which was dynamic based on performance. Participants were instructed to press a button with their right thumb for Go stimuli. In Block 1 (Standard Condition), trial #3 is a correct nonresponse to a No-Go stimulus resulting in +100 points and the stimulus duration/response window being shortened by 50 ms for subsequent trials. Trial #5 is an incorrect response to a No-Go stimulus resulting in −10 points, and the time window being increased by 50 ms

The Go/No-Go task was administered using E-Prime 3.0 (2016) software (Psychology Software Tools, Pittsburgh, PA) and resembled the task described in Lewis and colleagues (2006; Fig. 1). Lowercase letters were presented at the center of the screen, subtending a visual angle of approximately 2.86° (2° 51'). The stimuli were different for each block to reduce habituation without increasing difficulty. Block 1 presented “x” and “y,” Block 2 “o” and “p,” and Block 3 “u” and “d.” Participants were seated 60 cm away from the screen and instructed to press a response button on a custom, parallel port response device with their right thumb whenever a letter appeared (Go), unless it matched the letter from the previous trial (No-Go). Blocks 1 and 3 consisted of 200 trials, 33% of which were No-Go trials. Block 2 differed in that only 150 trials were presented (27% No-Go trials). The stimulus duration was determined based on participant performance. During Blocks 1 and 3, the duration was initially 700 ms; correct No-Go trials would result in a subtraction of 50 ms from the duration of the next stimulus, and a false-alarm response would add 50 ms to the duration of the next stimulus. Correct No-Go trials were worth +100 points, incorrect No-Go trials were worth −10 points, and missed Go trials were worth −5 points. Block 2 was designed to be more challenging than Blocks 1 and 3 by awarding only +15 points for correct No-Go responses and −150 points for incorrect No-Go responses, as well as adding only 30 ms to the response window for incorrect No-Go responses and subtracting 60 ms for correct No-Go trials. Similar to the task used in Lewis et al. (2006), Block 2 was intended to disproportionately induce negative emotions due to the change in difficulty and task parameters discussed above. Participants were shown a large red bar in the middle of the screen for any incorrect responses/omissions and were given score updates at pseudo-random intervals (on average, every 14 trials) throughout the task.

### Behavioral metrics: Signal Detection, mean response time, and response time standard deviation

We characterized behavioral performance (Table 2) by using the signal detection metric d-prime (d′; Stanislaw & Todorov, 1999). Typically thought of as a measure of individual discrimination of targets relative to non-targets, d′ considers hit-rate and false-alarm rate:

**Table 2:** Study measures (n = 106)

	M	SD	M	SD
**MPQ-155**				
PEM	52.9	8.7		
NEM	56.7	12.6		
CON	41.9	8.2		
Absorption	58.1	8.8		
**d′**				
Block 1 (Standard)	1.44	0.31		
Block 2 (Challenging)	1.30	0.40		
Block 3 (Standard)	1.53	0.38		
**GNG % Accuracy**	**Go**		**No-Go**	
Block 1 (Standard)	86.9	8.7	58.5	8.8
Block 2 (Challenging)	88.2	7.5	50.7	11.3
Block 3 (Standard)	86.9	8.3	61.8	10.3
**GNG Response Time (ms)**	**Go**		**No-Go Errors**	
Block 1 (Standard)	340.9	36.3	326.4	37.6
Block 2 (Challenging)	319.7	34.0	310.3	34.9
Block 3 (Standard)	312.8	38.4	310.5	42.3

Descriptive statistics for individual difference measures and Go/No-Go (GNG) task performance across the three task blocks. MPQ-155 subscales (PEM, NEM, CON, and Absorption) are reported as T-scores based on normative data. Task performance is indexed by signal detection sensitivity (d′), percent accuracy for Go and No-Go trials, and mean response time for correct Go responses and No-Go errors. Block 2 represents the most challenging condition. Block 2 d′ was significantly lower than Blocks 1 and 3 (*p* <.001), and Block 3 d′ was greater than Block 1 (*p* =.034). No-Go response error RTs were significantly faster than correct Go trials RTs (*p* <.001), and RTs were significantly faster in Blocks 2 and 3 compared with Block 1 (*p* <.001)

$${d}^{\prime}={\phi }^{-1}\left(hit\ rate\right)-{\phi }^{-1}\left(false\ alarm\ rate\right)$$

The function $\phi$(*x*) refers to the cumulative distribution function of the standard normal distribution, which gives the probability that a standard normal variable is less than or equal to x. The inverse function ${\phi }^{-1}$ gives the corresponding z-score for a given probability. Thus, a higher d′ score is indicative of an increase in the ability to detect targets (in this case, Go trials) embedded within nontargets (No-Go trials).

We also calculated the mean response time (RT) for all correct Go trials, and all incorrect No-Go trials (i.e., false-alarm trials), as well as response time standard deviation (RT SD). Mean RT is often used to assess processing efficiency, cognitive load, or control engagement, especially in tasks with high demands on attention or conflict resolution (Botvinick et al., 2001; MacDonald et al., 2000) and has been shown to correlate with FMT during cognitive control tasks (Cavanagh & Frank, 2014). RT SD offers a sensitive index of cognitive stability and may reflect the momentary fluctuations in attentional control that are relevant for understanding individual differences in top-down regulatory processes (Gallagher et al., 2015; Klein et al., 2006; Kofler et al., 2013; Swick et al., 2013).

### EEG acquisition and processing

EEG data was recorded using a 128-channel BioSemi ActiveTwo system with Ag/AgCl electrodes in a radial layout, and with CMS and DRL electrodes providing active feedback stabilization. Data were collected at a sampling rate of 2,048 Hz and initially referenced offline to bilateral earlobe electrodes to reduce common noise. Electrode offsets were kept between −30 to 30 mV (Kappenman & Luck, 2010). EEG data were imported into a custom independent component analysis-based (ICA) processing toolkit called ICAcleanEEG (Kang et al., 2015), and downsampled to 256 Hz. To prevent violation of the Nyquist frequency and suppress high-frequency noise, a low-pass filter with a 110 Hz cutoff was applied before downsampling. Additionally, the data were high-pass filtered at 0.5 Hz to reduce slow drifts from sweat, skin potentials, and movement, thereby improving ICA performance. The continuous EEG data were visually inspected to identify problematic electrodes and time segments. Electrode quality was first evaluated using a correlation matrix indexing similarity with neighboring channels across time; electrodes showing persistently low correlations with their nearest neighbors and lacking discernible neural signal were manually identified and removed for further preprocessing (mean = 3.6 electrodes; standard deviation [*SD*] = 2.7; range = 0–10). Their signals were restored by spherical spline interpolation after ICA denoising. In addition, a plot of maximum signal power across electrodes and time was inspected to detect disjointed recording segments (e.g., pauses and resumptions) and periods of high-amplitude, low-frequency noise indicative of poor electrode–scalp contact. After initial visual inspection, the trial data was epoched from −200 ms before the letter appeared to 1,000 ms after the letter appeared. These epochs were then subjected to ICA using the Fast ICA algorithm (Hyvärinen & Oja, 2000). We used a probabilistic ICA approach (Schwarz, 1978), applying a principal component analysis before ICA to reduce EEG dimensionality. This step addresses the mismatch between the number of electrodes and the true number of independent sources, which can otherwise yield mixed or unstable independent components. By reducing the data to its intrinsic dimensionality, principal component analysis improves the subsequent ICA decomposition. We used a maximum likelihood-based Bayesian approach for objective intrinsic dimensionality estimation (Mouraux & Iannetti, 2009; Rajan & Rayner, 1997). ICs were classified automatically, then manually inspected and reclassified as needed to either brain or noise-dominant, which was based on activity in the time series, voltage scalp topographies, power spectrum, and stimulus-related activity. On average, 40.2% of data variance (*SD* = 20.4; range = 6.4–84.6) was removed by excluding noise ICs. Of this 40.2% of the removed variance, an average of 17.4% was attributable to eye-related ICs (blinks and/or horizontal eye movements), 10.8% to muscle ICs, 10.8% to bad electrode ICs, and the remaining 1.2% to heartbeat, slow-wave, and other, not clearly identifiable, non-physiological artifacts. We then reconstituted the EEG after removing significant noise artifact ICs. After preprocessing, an average of 281.7 Go trials (*SD* = 28.5; range = 202–322) and 86.1 No-Go trials (*SD* = 13.5; range = 59–118) were included in the analysis. The artifact-reduced data were then re-referenced to the average signal across all electrodes to minimize spatial bias.

### Time-frequency analysis

**Fig. 2:**
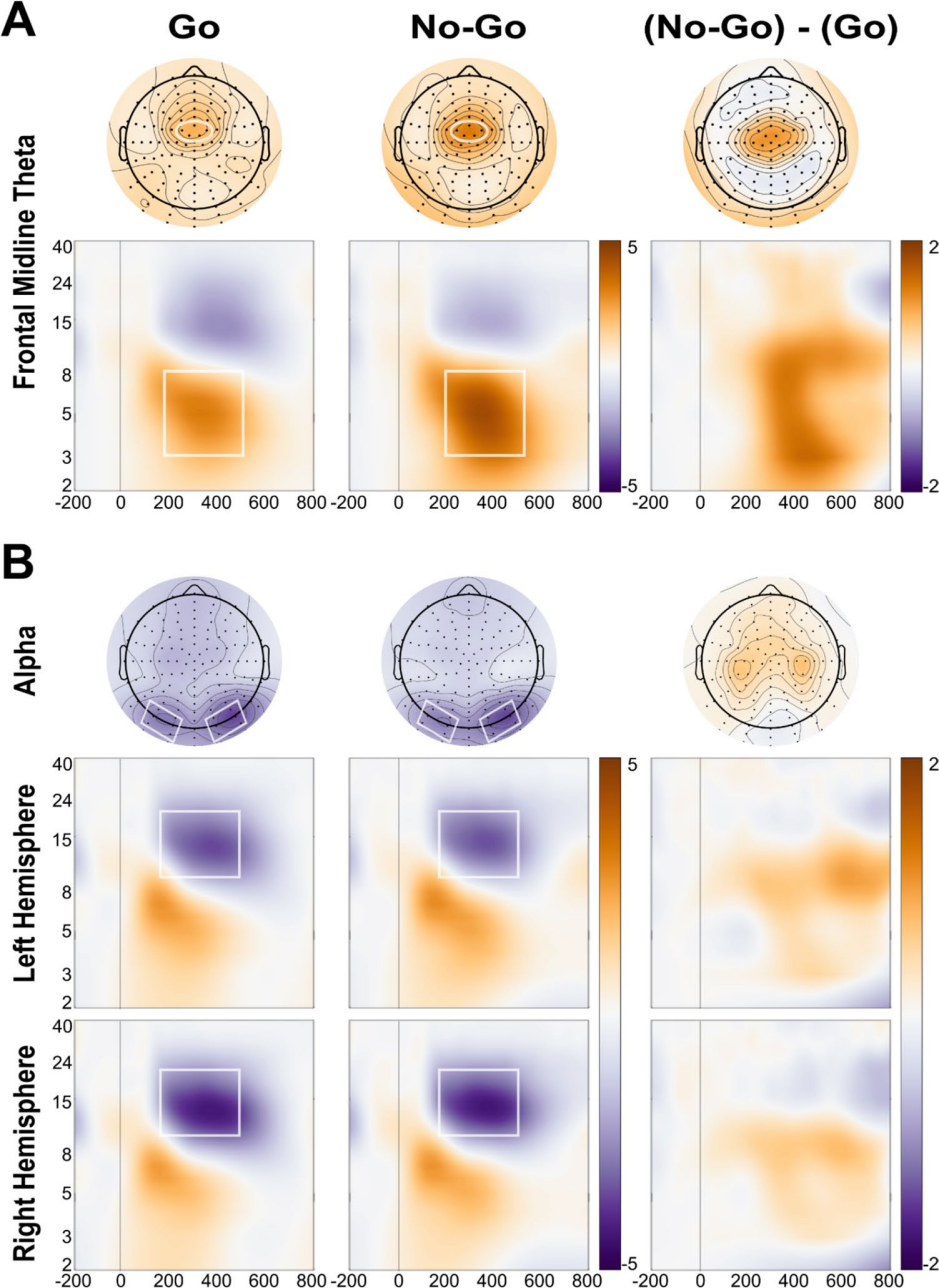
Time-frequency surface plots and corresponding scalp activation maps illustrating stimulus-locked spectral power across task conditions. **A)** Frontal midline theta power (3–8 Hz, dB), averaged over electrodes surrounding FCz (circled), during Go and No-Go trials. The top row shows scalp topographies of mean theta power from 200–500 ms poststimulus. The bottom row shows corresponding time-frequency surfaces averaged across electrodes of interest; the white box denotes the analyzed window. **B)** Posterior alpha power (10–20 Hz, dB), averaged over bilateral occipital clusters. Scalp topographies reflect mean alpha power from 180–500 ms poststimulus. Time-frequency plots below show alpha desynchronization, with boxed regions indicating the time–frequency window used in statistical analysis. The bilateral alpha band of the subtraction topography between No-Go and Go may reflect the button presses in the correct Go trials

Time-frequency analysis was performed using EEGLAB’s *newtimef* function (Makeig, 1993). A family of Morlet wavelets were convolved with single trials of EEG data. Wavelets ranged from 3 cycles in width at the lowest frequency of interest to 8 cycles in width at the highest frequency of interest, resolving 39 frequencies from 2 to 40 Hz with a linear spacing of 1 Hz. Time-frequency decompositions were computed at the single-trial level using EEGLAB’s *newtimef* function. For each participant, power estimates were averaged across trials within each condition (e.g., Go vs. No-Go, by block). The resulting condition-level averages were baseline-corrected relative to a prestimulus interval of −200 to 0 ms and converted to decibel (10×log_10_[power/baseline]) units before being entered into statistical analyses (Cohen, 2014). Based on previous literature, we generated a scalp topography of 3–8 Hz activity from 200 to 500 ms poststimulus to determine where theta frequency responses were maximal (Fig. 2). A cluster of five electrodes (BioSemi labels C1, C2, C23, D1, & D2) around electrode location FCz were chosen for total power analysis of FMT. We opted to analyze total power to capture FMT in a way that would be most comparable to most studies of FMT and anxiety-related traits (Cavanagh et al., 2017). Based on visual inspection of grand average time-frequency surfaces, we confirmed the appropriateness of examining theta power data for each trial type and each block at the FCz cluster from 200 to 500 ms poststimulus.

Additionally, the visual inspection of the time-frequency surfaces revealed notable desynchronization—primarily in the alpha band but extending into the low beta band. A topography of frequency power between 10 and 20 Hz from 180–500 ms poststimulus was generated to determine where this desynchronization was maximal (Fig. 2). We selected two separate electrode clusters around PO7 (BioSemi labels A9, A10, A11, A14, A15, & A16) and PO8 (BioSemi labels A27, A28, A29, B6, B7, & B8) and generated surfaces for each block and trial type at these electrodes. We subsequently extracted spectral power in the 10 to 20 Hz range during the 180 to 500 ms poststimulus interval. We primarily focused on theta and alpha brain activity corresponding to successful target detection and inhibition, so only correct Go and No-Go trials were analyzed. Time-frequency activity was visually examined across a broad frequency range; no consistent or interpretable modulation was observed in other bands, such as delta or gamma. Therefore, these other frequencies were not quantified.

### Data analyses

IBM SPSS Statistics v26 was used for all statistical analyses. To model the associations between behavioral performance, time-frequency data, and MPQ subscale scores, we used mixed effects models (MIXED) with restricted maximum likelihood estimation. For the models that included task-based time-frequency or RT data as dependent variables, we used repeated-measures fixed effects of Trial Type (Go, No-Go) and Block (first, second, third), with a diagonal covariance structure. The models of the signal detection metric d′ included only the Block fixed effect. All within-subjects post-hoc testing was evaluated using Bonferroni correction for multiple comparisons. We also included random effects of intercept due to improved Akaike information criterion and Bayesian information criterion model fit performance. All continuous variables entered into the mixed effects models—including the four MPQ scales (PEM, NEM, CON, and Absorption), brain measures, and behavioral performance indices (d′, mean RT, and RT SD)—were Z-scored prior to analysis. To show the directionality of significant MPQ associations, we reported standardized predictor coefficients with 95% confidence intervals. We examined simple slopes for any significant interaction effects. Lastly, indirect effects parallel mediations (Model 4) were run using the SPSS PROCESS macro (Hayes, 2017) to determine if FMT or lateral occipital desynchronization (collapsed across blocks and trial type) better explained the relationships between personality traits and d′ or RT task performance. In other words, we evaluated which neural process could account for the independent associations between MPQ traits (especially NEM) and subsequent disruptions in cognitive control. Each of the four MPQ traits were entered simultaneously as antecedents. All study effects were evaluated at ɑ =.05, and we performed 5,000 iterations of bootstrap resampling to estimate the *ab* indirect effect. Although not strictly required to interpret a significant indirect effect, we also reported results for the individual *a* and *b* paths to depict the directions of the associations. For consistency and ease of interpretation, only standardized coefficients are reported for the *a* and *b* paths.

## Results

### Target discrimination (d′)

There was a main effect of Block on target discrimination performance as measured by d′, *F*(2, 118) = 19.3, *p* <.001 (Table 2). Post-hoc testing revealed that this effect was driven primarily by a significant decrease in d′ for block two (*p*s <.001), as well as higher d′ in block three compared to block one (*p* =.034). The decline in performance during block two likely reflects the increase in task difficulty due to less time being added to the response window after No-Go errors, whereas the increase in performance during block three could potentially be explained by practice effects. Additionally, we noted a between-subjects main effect of NEM, *F*(1, 102) = 8.63, *p* =.004. Participants who scored higher on NEM had lower d′ regardless of task block (β = −.234, 95% confidence interval [CI] [−.392, −.076]). All other main effects and interactions of PEM, CON, and Absorption were not significant (*p*s >.063).

### Mean response time (RT)

There was an effect of Trial Type (Go, No-Go) on mean RT, *F*(1, 459) = 28.11, *p* <.001, an effect of Block, *F*(2, 279) = 61.30, *p* <.001, and an interaction between Block and Trial Type, *F*(2, 279) = 3.94, *p* =.021 (Table 2). Post-hoc testing of the main effect of Trial Type revealed that No-Go response errors were faster than Go RTs when averaged across blocks. The main effect of Block was driven by faster RT during blocks two and three compared with block one (*p*s <.001). Probing the interaction between Trial Type and Block, we found that during blocks one and two RTs for No-Go errors were faster than Go RTs, (*p*s <.001), but did not differ during block three (*p* =.459). Within the same model, we also saw a main effect of CON on mean RT, *F*(1, 102) = 6.00, *p* =.016, such that higher scores on CON led to increased RT regardless of the Trial Type or block (β =.201, 95% CI [.038,.364]). All other main and interaction effects for PEM, NEM, CON, and Absorption were not significant (*p*s >.194).

### Response time standard deviation (RT SD)

For RT SD, there was an effect of Trial Type, *F*(1, 429) = 368.1, *p* <.001, an effect of Block, *F*(2, 302) = 51.44, *p* <.001, and an interaction between Block and Trial Type, *F*(2, 302) = 6.08, *p* =.003 (Table 2). Follow-up analyses of this interaction showed that No-Go error RT SD was less than Go RT SD for all three blocks (*p*s <.001), but RT SD for Go did not differ between blocks one and three (*p* =.313). This model also revealed a between-subjects main effect of NEM on RT SD, *F*(1, 107) = 4.44, *p* =.038. Individuals who scored higher on NEM showed more variable RTs regardless of Trial Type or Block (β =.139, 95% CI [.008,.271]). Finally, we also found a three-way interaction between CON, Trial Type, and Block, *F*(2, 302) = 3.44, *p* =.033. Follow-up simple interaction between CON and Trial Type was significant only within block three (β = −.189, 95% CI [−.364, −.013]). Simple slopes for CON at Go and No-Go during block three were not significant (*p*s ≥.051). Additional simple interactions within Blocks 1 and 2 were not significant (*p*s ≥.111). Therefore, associations with CON were not considered further. There were no other interaction effects for PEM, NEM, CON or Absorption (*p*s >.087).

### Frontal midline theta

**Fig. 3:**
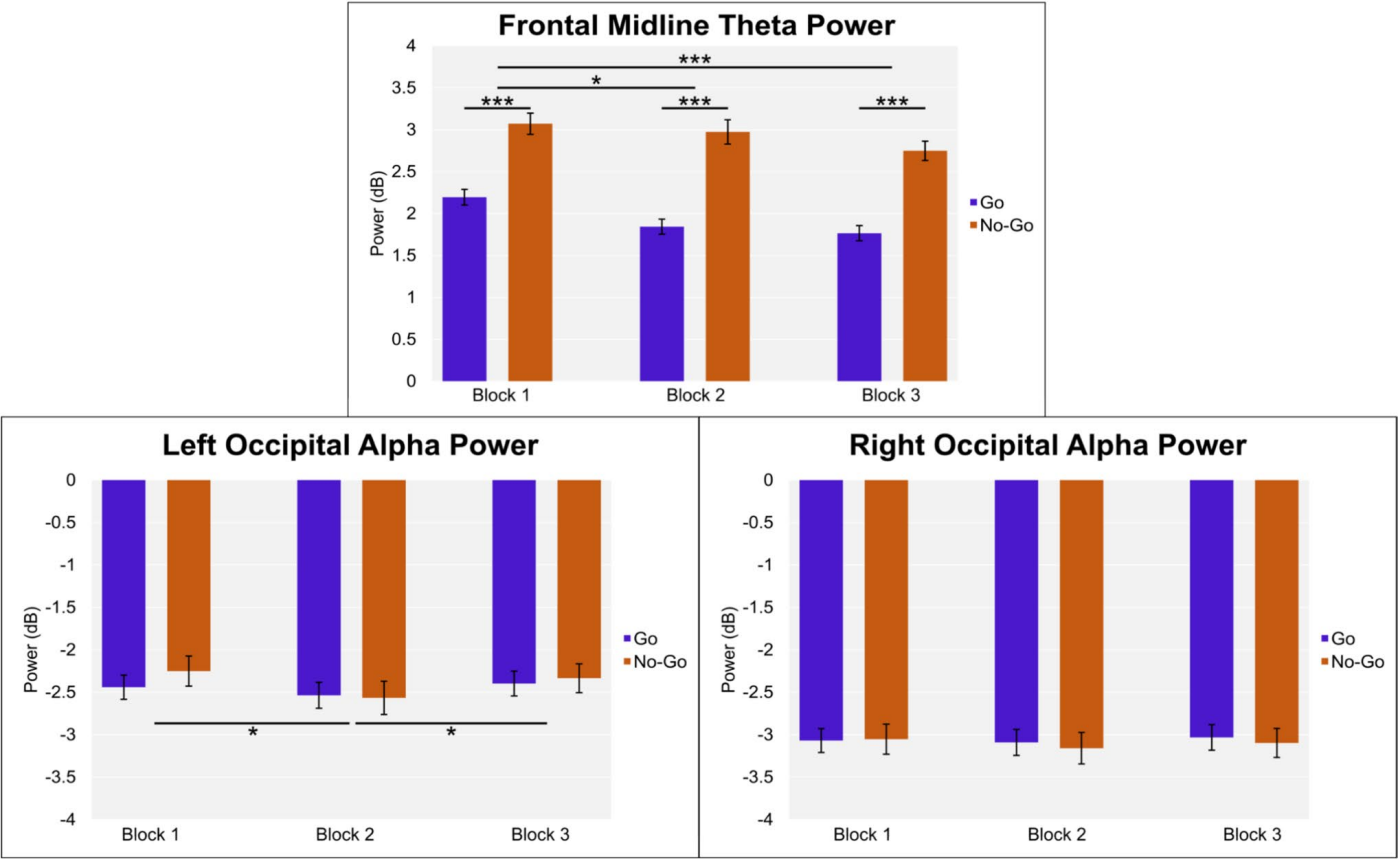
Estimated marginal means of FMT and left and right Occipital Alpha power (dB) for Go and No-Go Trial Types across each block. **A)** FMT power was greater for No-Go trials compared to Go trials across all three blocks. FMT power was also greater during Block 1 relative to Blocks 2 and 3. **B)** Occipital Alpha desynchronization was greater in the right occipital region relative to the left. Occipital Alpha desynchronization was greater during Block 2, driven mostly by differences in the left occipital region. *p <.05; **p <.01; ***p <.001

Our analysis revealed an effect of Trial Type *F*(1, 327) = 243.55, *p* <.001 on FMT (Figs. 2 and 3). As expected, frontal midline theta (FMT) power was greater for correct No-Go trials compared with correct Go trials reflecting greater need for cognitive control. There was also an effect of block on FMT *F*(2, 268) = 13.68, *p* <.001. Frontal midline theta power decreased from Block 1 to 2 (*p* =.024) and from block one to three (*p* <.001), but Blocks 2 and 3 did not differ (*p* =.166). Similar to the d′ findings, this general decline in FMT from block to block may be explained by habituation, cognitive fatigue, and/or task mastery. There was no interaction between Trial Type and Block (*p* =.319).

Within the same model, we saw main effects of NEM *F*(1, 115) = 4.42, *p* =.038 and Absorption *F*(1, 115) = 4.51, *p* =.036. Individuals who scored higher on NEM showed attenuated FMT (β = −.149, 95% CI [−.29, −.001]). This result parallels our finding between NEM and d′. Individuals who scored high on Absorption also showed attenuated FMT (β =−.152, 95% CI [−.293, −.01]). All other main and interaction effects for PEM, NEM, CON, and Absorption were not significant (*p*s >.149).

### Occipital alpha

There was a significant effect of Block on Occipital Alpha desynchronization, *F*(2, 643) = 3.57, *p* =.029 (Figs. 2 and 3). Post-hoc testing indicated that desynchronization was significantly greater in Block 2 compared with Block 1 (*p* =.045), with a trend-level increase relative to Block 3 (*p* =.085). This finding supports existing literature suggesting that alpha desynchronization increases with increasing task difficulty. We also saw an effect of Laterality *F*(1, 978) = 215.64, *p* <.001, where alpha desynchronization was more pronounced in the right occipital cluster compared with the left. Furthermore, we observed significant interactions between Laterality and Absorption *F*(1, 978) = 9.99, *p* =.002, as well as Laterality and CON *F*(1, 978) = 4.55, *p* =.033, but the simple slopes analyses failed to be significant (*p*s ≥.329). There were no other PEM, NEM, CON, or Absorption significant main effects or interactions (all *p*s ≥.054).

### Mechanistic role of neural responses on the relationship of negative emotionality and absorption to cognitive control

**Fig. 4:**
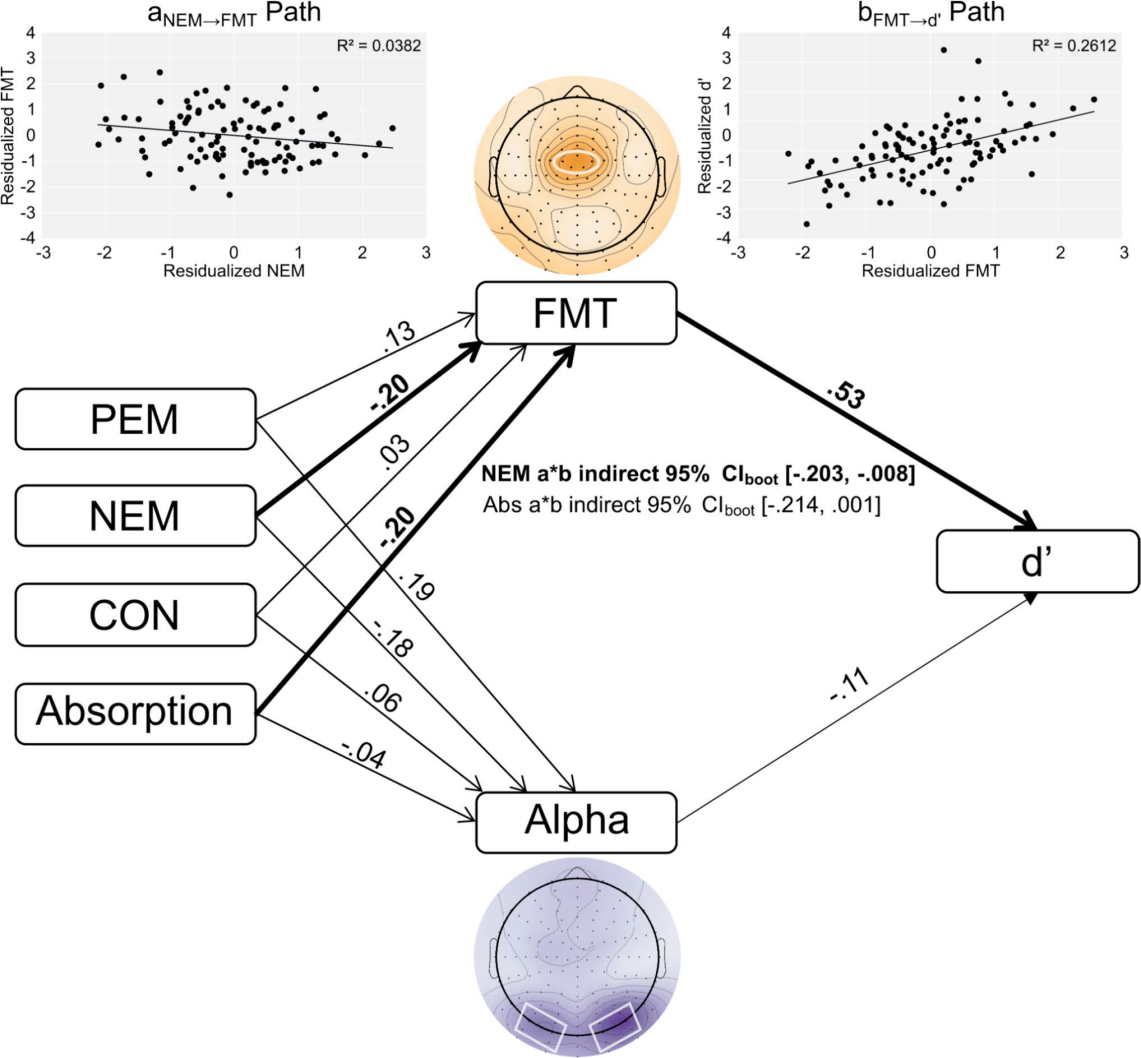
Parallel mediation model illustrating indirect effects between MPQ scales, Positive and Negative Emotionality (PEM, NEM), Constraint (CON), and Absorption, and task performance (d′), mediated by frontal midline theta (FMT) and occipital alpha power. Standardized beta coefficients are displayed on each path; bolded values and arrows indicate statistically significant effects. FMT and alpha power were averaged across all conditions within their respective frequency bands of interest (3–8 Hz for FMT, 10–20 Hz for alpha). Topographic plots depict spectral power at selected electrode clusters. Scatter plots show associations between residualized variables for the a-path (NEM to FMT) and b-path (FMT to d′)

Lastly, we ran mediation models to investigate whether FMT and/or occipital alpha could explain the independent associations between NEM and task performance as measured by d′, mean RT, and RT SD (Fig. 4). We found that the relationship between NEM and d′ was mediated by FMT (*ab* = −.105, 95% CI_boot_ [−.207, −.009]), but not alpha desynchronization (*ab* =.02, 95% CI_boot_ [−.012,.093]). Within this model, there were significant associations between NEM and FMT (*a* = −.200, 95% CI [−.021, −.0001]) as well as FMT and d′ (*b* =.527, 95% CI [.109,.217]) (Fig. 4). The relationship between NEM and mean Go RT was also mediated by FMT on correct Go trials (*ab* =.071, 95% CI_boot_ [.001,.168]), but not alpha desynchronization (*ab* = -.011, 95% CI_boot_ [-.067,.028]). In this model, the relationship between NEM and FMT on correct Go trials was negative, but not significant (*a* = -.194, 95% CI [−.019,.0003]). However, the relationship between FMT and Go RT was significant (*b* = −.366, 95% CI [−20.539, −6.39]). For RT SD, FMT from Go trials mediated the association between NEM and Go RT SD (*ab* =.075, 95% CI_boot_ [.001,.167], but alpha desynchronization did not (*ab* = −.011, 95% CI_boot_ [−.072,.026]). Within the same model, the negative association between NEM and FMT on correct Go trials was not significant (*a* = −.194, 95% CI [−.0191,.0003]), but there was a significant relationship between FMT and Go RT SD (*b* = −.387, 95% CI [−10.796, −3.71]). Thus, similar indirect effects from NEM through FMT could explain disruptions in three separate behavioral metrics in cognitive control.

We also examined the independent associations of Absorption in the same mediation model. The indirect effect between Absorption and d′ was not significantly mediated by FMT (*ab* = –.106, 95% CI_boot_ [–.214,.001]) or by occipital alpha desynchronization (*ab* =.005, 95% CI_boot_ [–.023,.036]). Within this model, there was an association of similar magnitude as NEM between Absorption and FMT (*a* = –.203, 95% CI [–.147, –.002]). For mean Go RT, neither FMT (*ab* =.064, 95% CI_boot_ [–.007,.152]) nor occipital alpha desynchronization (*ab* = –.001, 95% CI_boot_ [–.025,.020]) significantly mediated the relationship between Absorption and performance. Similarly, the relationship between Absorption and Go RT SD was not mediated by FMT (*ab* =.068, 95% CI_boot_ [–.007,.166]) or by occipital alpha desynchronization (*ab* = –.001, 95% CI_boot_ [–.023,.024]). All other indirect effects models using PEM and CON as the antecedent were not significant.

## Discussion

The present study examined the relationship between frontal midline theta (FMT) power and trait negative emotionality (NEM) within the context of a Go/No-Go cognitive control task. Consistent with our hypotheses and prior research, we observed that FMT power was significantly greater during correct No-Go trials compared to correct Go trials. There was also a significant increase in alpha desynchronization during the second, most difficult, block of the task, and alpha desynchronization was greater overall in the right occipital cluster compared to the left. We identified simultaneous main effects of both NEM and Absorption on FMT power, where individuals with higher levels of NEM and Absorption exhibited reduced FMT power. Parallel mediation analysis indicated that FMT facilitated an indirect effect between NEM and d′. Individuals with higher NEM scores, which reflect greater emotional reactivity and elevated risk for psychopathology, showed poorer task performance in part due to reduced FMT power. This mediating role of FMT was also observed for two complementary indices of cognitive performance: mean reaction time (RT) and reaction time variability (RT SD). This suggests that individuals high in NEM not only performed less accurately but also responded more slowly and variably, in part due to diminished midline frontal cognitive control. Conversely, FMT did not mediate the relationship between Absorption and d′. Together, these findings demonstrate that individual differences in the personality traits NEM and Absorption are associated with variation in FMT power during a challenging cognitive control task, suggesting a potential neurophysiological mechanism linking personality to transdiagnostic vulnerability for psychopathology.

The main effect of NEM on FMT power extends our understanding of how enduring personality traits associated with psychopathology risk are reflected in neural markers of cognitive control. Individuals with elevated trait negative emotionality may have a diminished capacity to effectively engage these cognitive processes, particularly under stress. This may contribute to poorer real-world outcomes, including increased vulnerability to both internalizing and externalizing manifestations of psychopathology (Wolf et al., 2012). Supporting this idea, White et al. (2018) found that greater activation of the cognitive control network during an affective Stroop task was associated with fewer PTSD symptoms in a trauma-exposed military sample, suggesting that neural systems which support cognitive control may also serve in a protective role against stress-related disorders.

Meta-analytic evidence suggests that anxiety is often associated with increased FMT power, presumably reflecting compensatory upregulation of cognitive control mechanisms in response to action-outcome uncertainty (Cavanagh & Shackman, 2015). One possible reason our findings indicate the opposite pattern in individuals high in NEM is that the participant sample was largely without full-threshold manifestations of psychopathology that would have prevented the recruits from joining the military. Furthermore, by analyzing only correct Go and No-Go trials, we sought to reduce the influence of error-related control processes and isolate theta activity associated with successful performance and relatively certain behavioral outcomes. The attenuation of FMT power during response inhibition in individuals high in NEM may reflect a disruption in the functioning of neural resources supporting cognitive control during a challenging behavioral inhibition task with clear action-outcomes. It is also important to note that heightened FMT has been elicited by tasks involving punishment and conflict that were related to anxiety measured with instruments such as the State-Trait Anxiety Inventory and the Behavioral Inhibition System scale, which emphasize sensitivity to threat and punishment cues (Spielberger et al., 1983; Carver & White, 1994), while the MPQ-NEM scale captures a broader dispositional vulnerability encompassing stress reactivity, alienation, and aggression. Our findings also align with recent work by Buzzell et al. (2020), who showed that early psychosocial neglect predicted reduced error-related FMT and increased general psychopathology in adolescence. In their randomized controlled trial, FMT served as a mediating mechanism linking early adversity to broad transdiagnostic risk, suggesting that deficient theta modulation may be a core neurobiological marker of impaired self-regulation. Notably, theta power did not uniquely predict internalizing or externalizing symptoms once general psychopathology was accounted for by Buzzell and colleagues, emphasizing its role in domain-general vulnerability. Together with our findings, these results emphasize the relevance of FMT as a broadly applicable, transdiagnostic marker of cognitive-affective dysfunction.

This interpretation is also consistent with much neuroanatomical and functional imaging evidence implicating the anterior portion of the cingulate cortex (ACC) as a generator of FMT (Asada et al., 1999; Tsujimoto et al., 2006; Ishii et al., 1999), which drives affective evaluation, conflict monitoring, and executive regulation (Zhang & Peng, 2023). The ACC has been shown to integrate emotional salience with functions such as error monitoring, behavioral inhibition, and motivational adjustment (Bush et al., 2000; Devinsky et al., 1995; Botvinick et al., 2001; Holroyd & Coles, 2002). These processes are essential for successful performance on tasks like the Go/No-Go paradigm. High-NEM individuals, characterized by heightened emotional reactivity and stress sensitivity, may experience dysregulation in this system, impairing their capacity to engage adaptive control. Thus, the observed FMT attenuation may not reflect disengagement after initial over-engagement (as seen in some ERN findings, see Luu et al., 2000), but rather a general failure to recruit cognitive-affective control systems under the condition of sustained cognitive demand. This neural inefficiency may help to explain the poorer behavioral performance observed in high-NEM individuals, including not only reduced discrimination accuracy but also slower and more variable response times, and supports the potential utility of FMT as a neurobiological marker of transdiagnostic risk.

Although FMT has often been interpreted as an index of cognitive control, its conceptual status as a trait-like risk marker or as a state-dependent mechanism remains an open question. Evidence suggests that FMT exhibits moderate stability across sessions (Gold et al., 2013) and relates to enduring individual differences in traits such as anxiety and negative emotionality (Osinsky et al., 2017; Suetsugi et al., 2000), supporting its potential role as a vulnerability marker. At the same time, FMT is highly sensitive to task demands and emotional context, indicating that it also reflects state-dependent recruitment of control processes. Thus, FMT may best be viewed as a dynamic process with both stable and flexible components, which complicates simple trait–state distinctions and magnifies the importance of studying it across different contexts and timescales.

The observed decline in FMT power across blocks may reflect a combination of habituation, increased task mastery, task difficulty differences between blocks, and the effects of mental fatigue. Prior studies demonstrate that FMT is sensitive to task demands (Gevins et al., 1997; Zakrzewska & Brzezicka, 2014) and scales with cognitive effort (McFerren et al., 2021; Onton et al., 2005; Smit et al., 2005). Although sustained effort typically results in FMT decreasing over time (Arnau et al., 2021), such reductions are often accompanied by worse performance. In contrast, the present study revealed a decline in event-related FMT across blocks alongside improved accuracy and faster response times. This dissociation may be due to the block structure of the task, which increased in difficulty from Block 1 to Block 2, then decreased from Block 2 to Block 3. Thus, while time-on-task and cognitive fatigue increased steadily, cognitive effort peaked in Block 2 and then declined in Block 3. One interpretation is that early increases in FMT reflected heightened engagement with increasing demands, followed by a reduction in control signaling as participants adapted and automated their responses. Frontal midline theta has been proposed to serve as an alarm signal in response to surprising or control-relevant events, and our findings suggest that as a task becomes familiar and behavior becomes more efficient, the demands on neural functions supporting cognitive control diminish. Future studies may clarify these dynamics by counterbalancing block difficulty across participants to separate the effects of time-on-task from cognitive demand.

The negative association between Absorption and FMT power suggests that individuals high in this trait may allocate fewer neural resources to external tasks that require the engagement of cognitive control. Absorption is characterized by an increased capacity for immersion in experience, such as vivid imagery, intense fantasizing, and deep involvement in perceptual or imaginative processes (Lifshitz et al., 2019; Roche & McConkey, 1990). This alteration in attention may compete with the task-focused control processes typically elicited by FMT. Consistent with this interpretation, Miller et al. (2003) found that military veterans with PTSD, often characterized by dysregulated attention and heightened emotional responsivity, scored higher not only on NEM but also on Absorption. Furthermore, prior studies have shown that individuals high in Absorption exhibit increased reactivity to emotionally salient stimuli regardless of their valence, suggesting that attention is distributed generally rather than strategically in this context (Benning et al., 2015; Kreutz et al., 2008). This likely reflects the generalized emotional responsiveness associated with absorption that cannot be explained by processes specific to either NEM or PEM (Tellegen & Waller, 2008). During a task demanding sustained cognitive control like Go/No-Go, such nonspecific attentional deployment may impair the efficient recruitment of frontal control mechanisms, as reflected in reduced FMT.

The increase in occipital alpha desynchronization during Block 2, relative to Blocks 1 and 3, is consistent with prior research showing that alpha desynchronization is augmented as task demands increase (Boiten et al., 1992; Dujardin et al., 1993; Erickson et al., 2019). This pattern suggests that the heightened difficulty of Block 2 elicited greater engagement of visual-attentional systems. Although alpha desynchronization was stronger across trial types and blocks in the right hemisphere, consistent with right-hemispheric dominance in visuospatial attention (Shulman et al., 2010; DiNuzzo et al., 2022), the increase in desynchronization during Block 2 was primarily driven by changes in the left occipital cluster. This dissociation may indicate that both occipital hemispheres are engaged under peak cognitive load, with the left hemisphere exhibiting greater flexibility or responsiveness to increasing task demands. The absence of associations between occipital alpha desynchronization and either task performance or FMT suggests that alpha modulation in these regions may support perceptual or attentional adjustments that are functionally distinct from the frontal systems responsible for cognitive control and behavioral inhibition.

There are several limitations that should be acknowledged as it relates to these findings. First, although the mediation models are consistent with the idea that FMT links NEM to cognitive control performance, we cannot draw firm causal conclusions. However, the MPQ, which is designed to produce relatively stable estimates of trait emotionality, was administered prior to EEG data collection (53 days on average), providing some temporal separation between the predictor (NEM) and the neural and behavioral outcomes. This timing may strengthen the plausibility of associations between personality traits, task-related brain function, and performance. Even so, other variables could still account for the observed associations, such as state-level stress or anxiety on the day of data collection, task engagement, or other personality traits like conscientiousness. Second, the sample consisted exclusively of U.S. Army National Guard recruits, which may limit the generalizability to broader civilian or clinical populations. In addition, a larger sample size would enhance statistical power and might improve the capacity to detect more nuanced interactions between MPQ personality traits and task-related EEG responses. Replication in larger and independent samples is necessary to confirm the stability and generalizability of the observed effects, and rule out Type I error.

Furthermore, the Go/No-Go paradigm primarily targets response inhibition, working memory, and sustained attention, raising questions about whether the observed effects would extend to other domains of cognitive control (i.e., set shifting). The variant of Go/No-Go we used places additional demands on working memory more than other variants due to its N-back nature. This increased load on working memory relative to other Go/No-Go paradigms may limit the generalizability of our findings to other inhibitory control tasks with less working memory demands. By focusing exclusively on neural responses during correct trials, our analyses capture only one aspect of theta’s functional role, whereas other prior work has examined response-locked (ERN) and feedback-locked activity. Theta elicited by errors or feedback may reflect distinct neural mechanisms and exhibit different relationships with MPQ traits. It should also be acknowledged that some correct trials may have followed errors and could therefore include compensatory adjustments such as post-error slowing or momentary increases in control engagement (Stevens et al., 2025). However, this scenario was uncommon as No-Go trials could never precede other No-Go trials and errors on Go trials were infrequent. Also, while posterior alpha desynchronization did not mediate behavioral outcomes, its modulation across task blocks may reflect additional factors such as fatigue, motivation, or arousal, indicating a need for future work to determine state and trait influences on this neural activity. In addition, future studies could dissociate evoked and induced components of oscillatory activity to better characterize the relative contributions of phase- versus nonphase-locked theta dynamics to cognitive control.

## Conclusions

Overall, the results of the present study provide evidence that FMT activity serves as a neurophysiological mechanism linking trait NEM to cognitive control performance in a response inhibition task. Specifically, individuals with higher levels of NEM showed reduced FMT activity, which was linked to poorer task performance, slower reaction times, and increased reaction time variability, indicating that impairments in cognitive control may help explain how affective vulnerability is expressed as a behavioral deficit. Posterior alpha desynchronization was sensitive to task engagement across blocks but did not account for individual differences in performance, nor was it related to any of the MPQ primary trait scales. These results are important in understanding the integration of trait-level personality measures with task-evoked neural dynamics in the context of individual differences in self-regulation. Future research would benefit from a longitudinal analysis to clarify the temporal stability and causal direction of these associations. It would also be informative to incorporate clinical or at-risk populations to assess the translational relevance of FMT as a potential biomarker for affective dysregulation. Such efforts would also help determine whether FMT and its underlying neural generators represent viable targets for cognitive or neurofeedback-based interventions with the goal of improving regulatory control. Using a variety of cognitive control tasks and examining other neural markers may further clarify the broader cognitive and affective networks through which personality traits influence goal-directed behavior.

## Supplementary Information

Below is the link to the electronic supplementary material.

Supplementary file1 (DOCX 7197 KB)

## Data Availability

Data and materials can be made available upon request after completion of a Data Use Agreement.

## Code availability

All non-proprietary code can be made available upon request.
