# Supplementary material for: Neural mechanisms of negative emotionality and cognitive control: The role of frontal midline theta
Source: Cogn Affect Behav Neurosci. 2026 Apr 14;26(4):1538–55. doi: 10.3758/s13415-026-01434-0 (PMC13385006; doi:10.3758/s13415-026-01434-0)
Supplement: Supplementary file 1 — Supplementary file1 (DOCX 7197 KB) [file 13415_2026_1434_MOESM1_ESM.docx]

**Neural Mechanisms of Negative Emotionality and Cognitive Control: The Role of Frontal Midline Theta**

***Supplemental Materials***

**Follow-up Examination of Individual Negative Emotionality Primary Trait Scales**

The Multidimensional Personality Questionnaire (MPQ)-Brief Form (BF)/155 uses weighting equations to calculate the broad trait scales of Positive Emotionality (PEM), Negative Emotionality (NEM), and Constraint (CON). These equations incorporate scores from 10 of the 11 primary trait scales, but are influenced most strongly as follows: Wellbeing, Social Potency, Achievement, and Social Closeness for PEM; Stress Reaction, Alienation, and Aggression for NEM; Control, Harm Avoidance, and Traditionalism for CON. Given the observed association between frontal midline theta (FMT) and NEM in the main manuscript, we conducted follow-up analyses examining the primary trait scales that most greatly influence NEM scores.

We executed MIXED in SPSS with a random effect of subject similar as reported in the main manuscript. We conducted separate models for the three NEM-relevant primary trait scales: Stress Reaction, Alienation, and Aggression. For all models, we observed the expected significant within-subject associations for Trial Type and Block (qualitatively identical to the effects reported in the main manuscript). We observed a main effect of Alienation, *F*(1, 117) = 11.65, *p* < .001, which manifested as a negative relationship with FMT (β = -.228, 95% CI [-.360, -.096]). There were no significant interaction effects with Alienation (p*s* ≥ .263). The main effects of Stress Reaction (*p* = .127) and Aggression (*p* = .066), estimated in separate models, were not significant. We examined the simple slopes of those predictors for comparison with Alienation: both associations for Stress Reaction (β = -.106, 95% CI [-.244, .031]) and Aggression (β = -.128, 95% CI [-.264, .009]) were negative and in the same direction. There were no significant interaction effects with Stress Reaction (p*s* ≥ .308) or Aggression (p*s* ≥ .688).

Within our sample of new military recruits, the NEM-relevant alterations in FMT were most closely associated with Alienation (given to feeling maligned or mistreated). However, we observed similar, albeit attenuated and non-significant, associations with Stress Reaction (given to negative emotions) and Aggression (vindictive, enjoying aggression). The NEM broad trait score may be capturing the shared negative associations across the three primary trait scales. For future studies, it would be beneficial to further explore the specificity of this FMT association with Alienation as opposed to NEM more generally.

**Figure S1.** Midline Time-frequency Surfaces by Block.


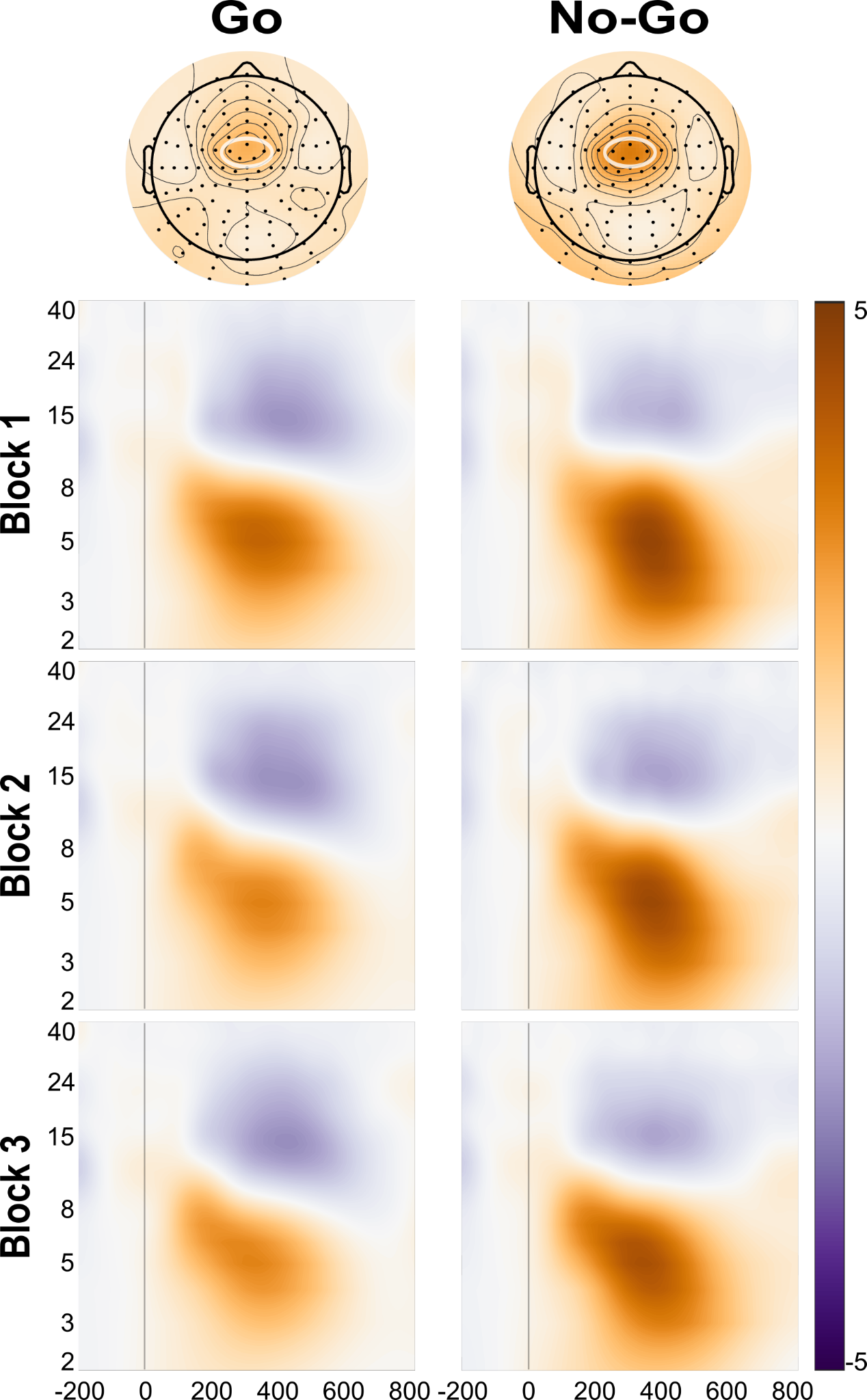


**Note.** Time-frequency surfaces depicting ERSP (dB) of frontal midline theta for each Block and both Trial Types at a cluster of frontal midline electrode sites. Data depicted is derived from correct trials only.

**Figure S2.** Bilateral Posterior Time-frequency Surfaces by Block.


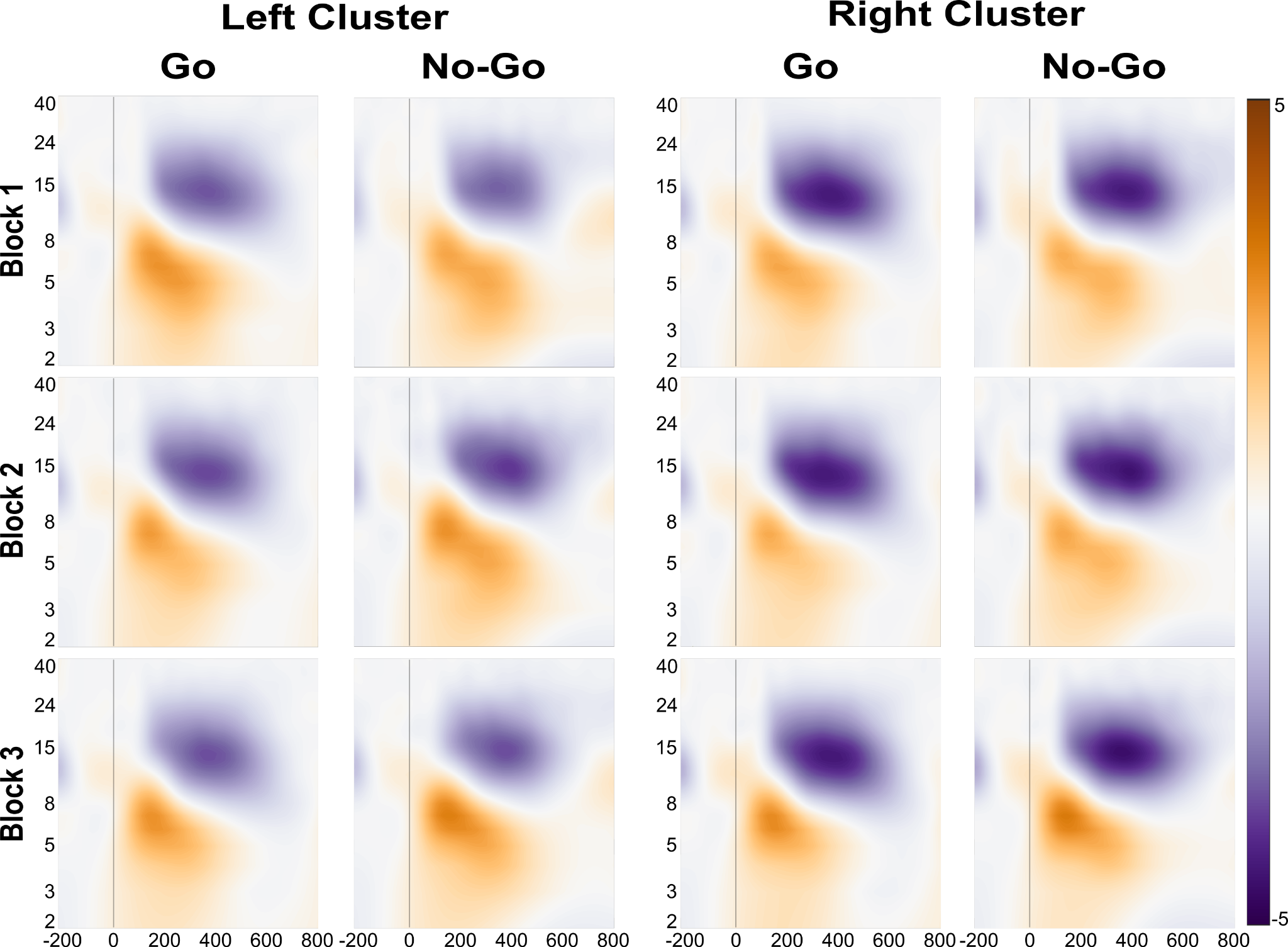


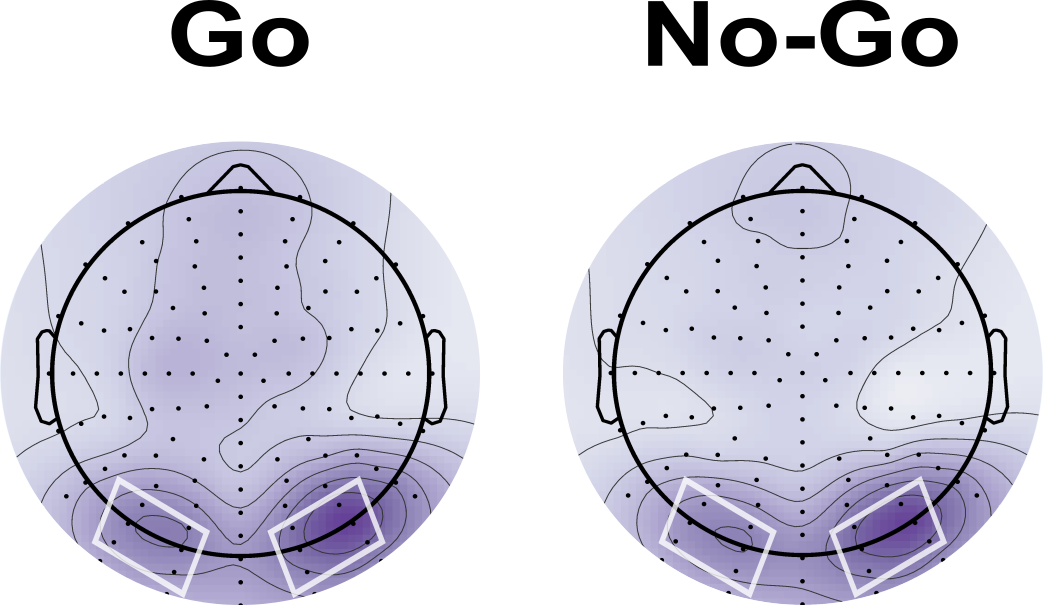


**Note.** Time-frequency surfaces depicting ERSP (dB) of occipital alpha for each Block and both Trial Types at clusters of left and right occipital electrode sites.


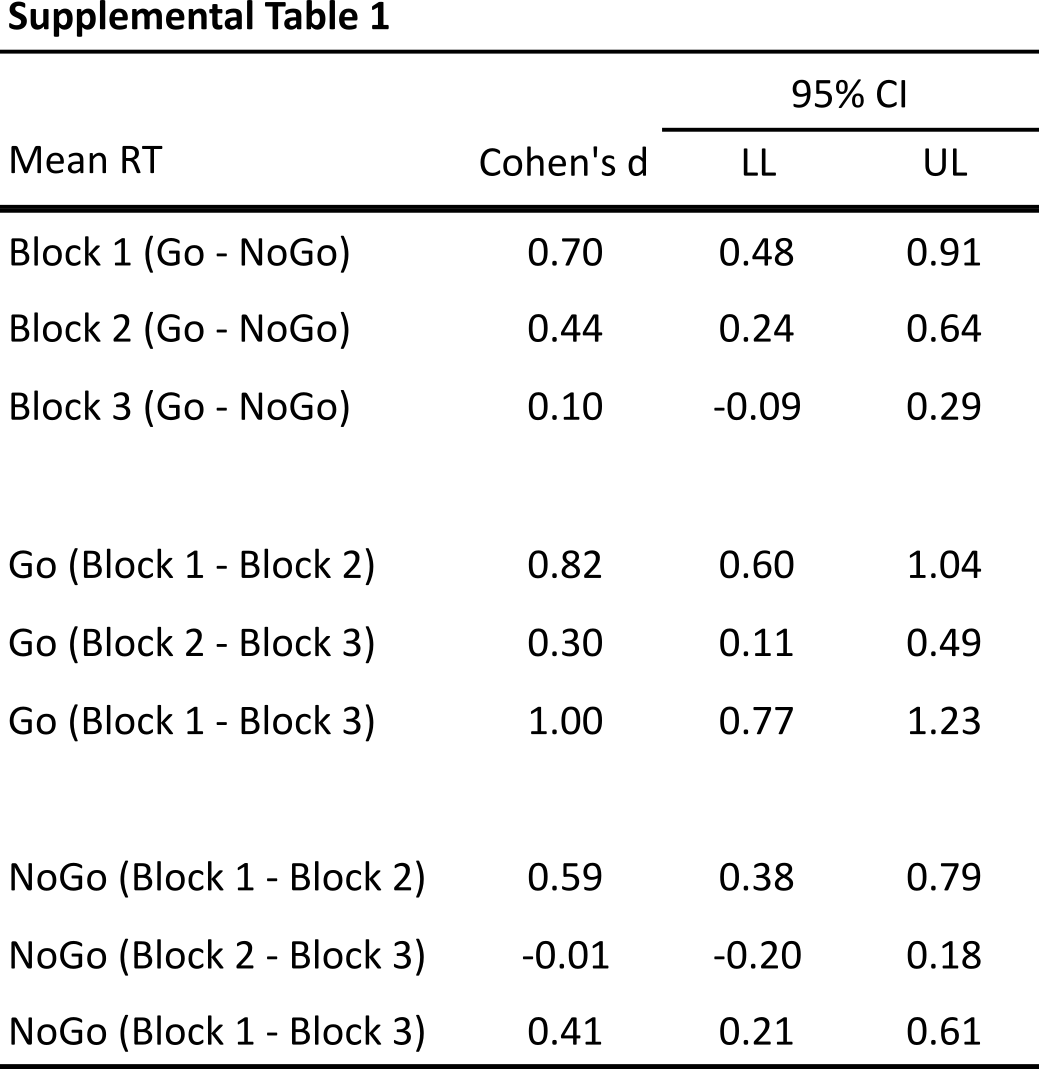


**Note.** Results of paired samples t-test for mean response time (RT) with Cohen’s d for each test.


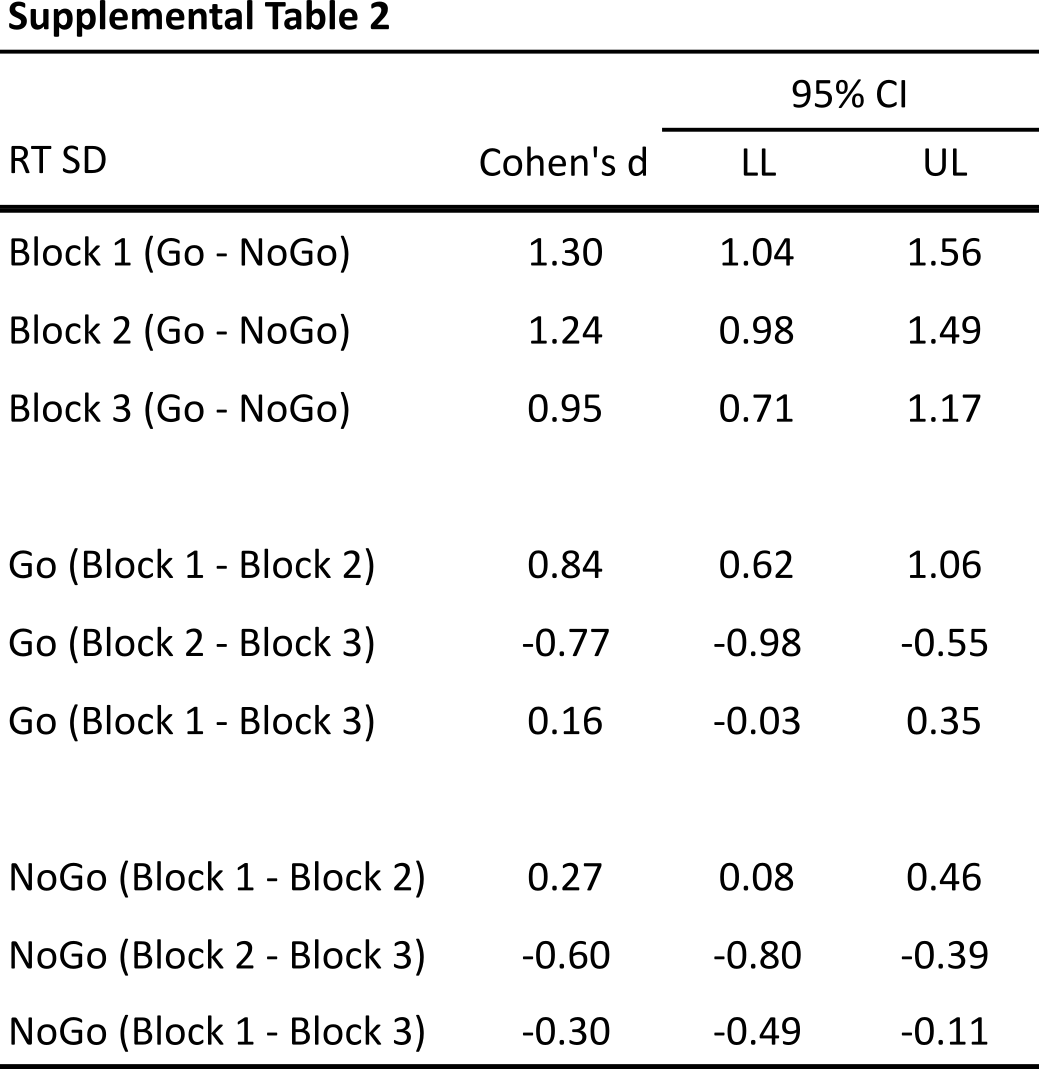


**Supplemental Table 2.** Results of paired samples t-test for RT standard deviation (RT SD) with Cohen’s d for each test.


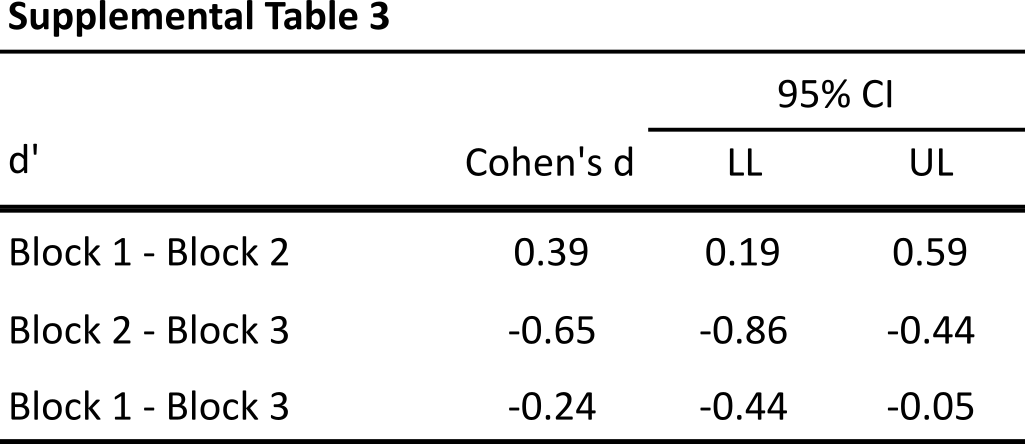


**Supplemental Table 3.** Results of paired samples t-test for d′ with Cohen’s d for each test.


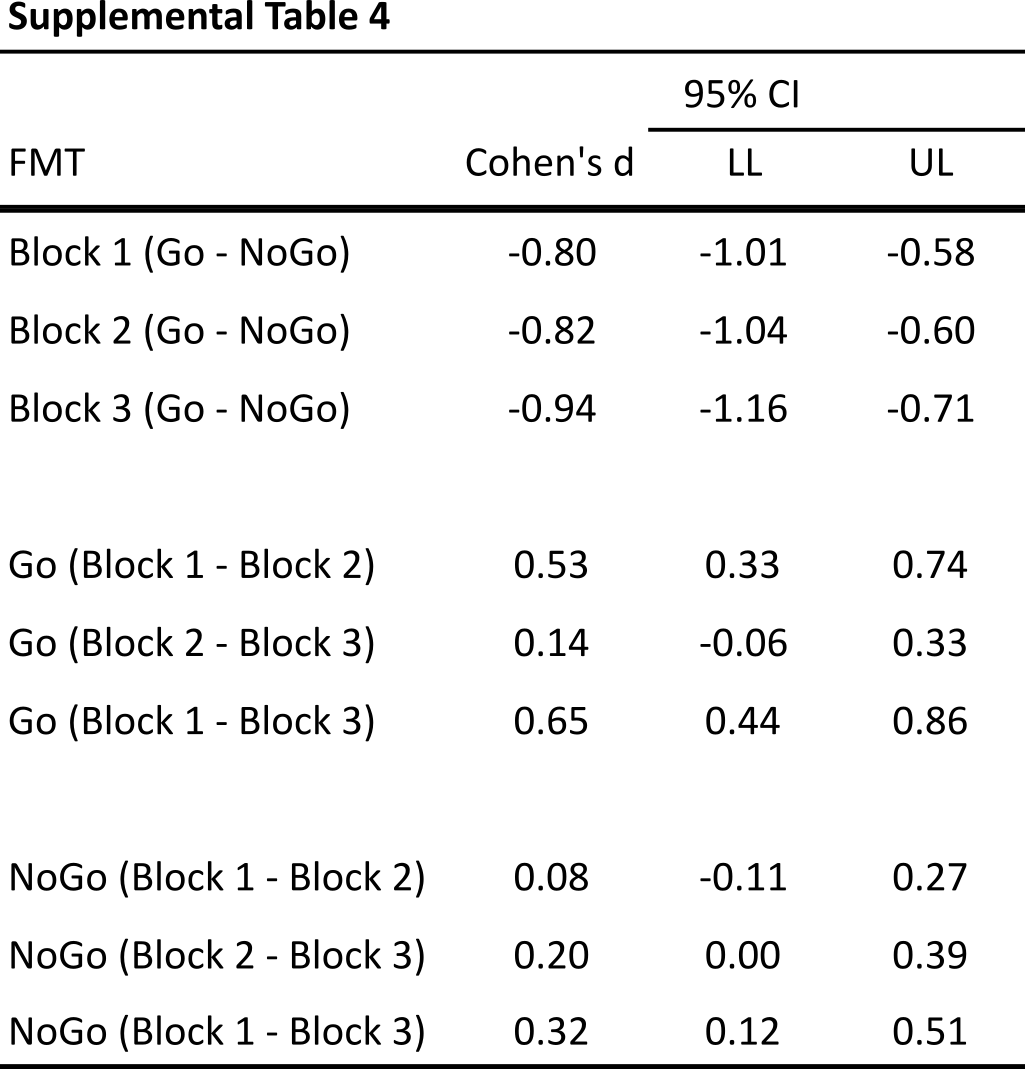


**Supplemental Table 4.** Results of paired samples t-test for frontal midline theta (FMT) with Cohen’s d for each test. Included data is from correct trials only.


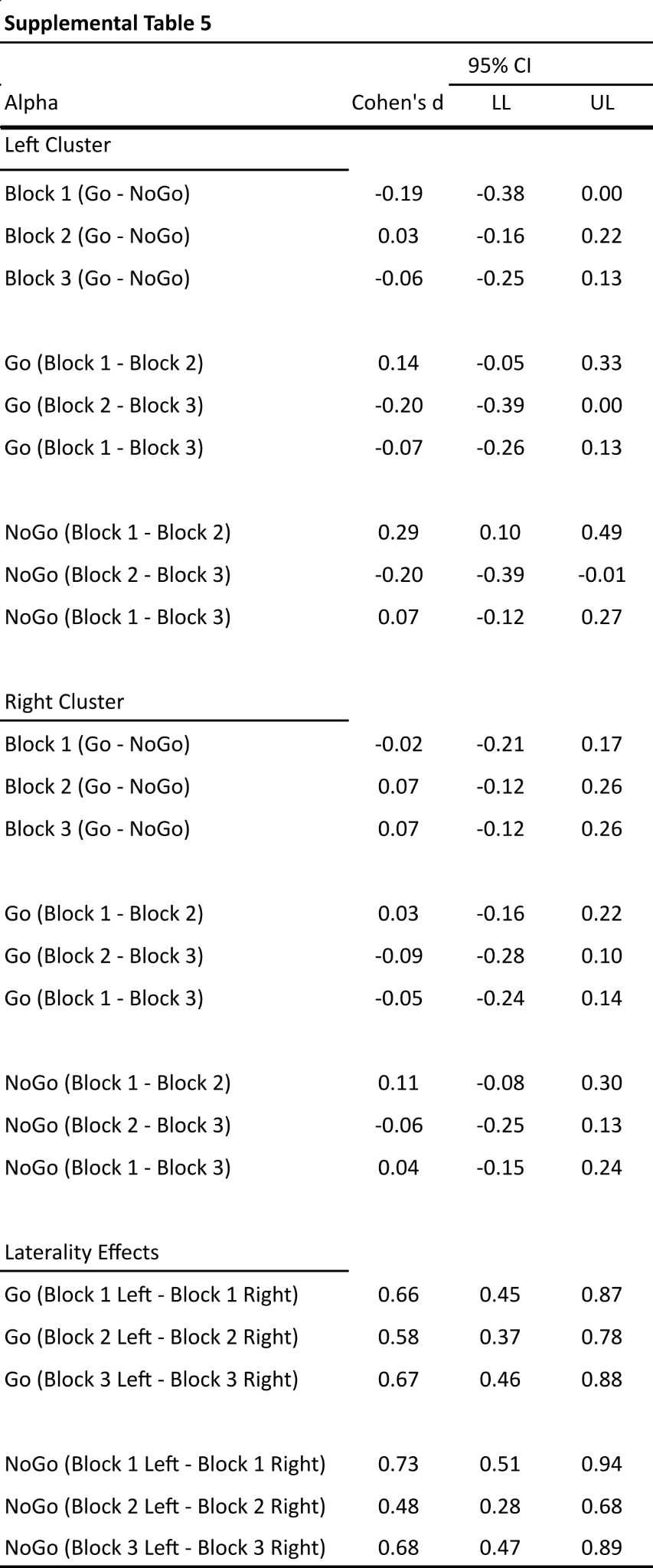

**Supplemental Table 5.** Results of paired-samples t-tests for occipital alpha power, with Cohen’s d values indicating effect sizes. Analyses include correct trials only; decreases in alpha power reflect task-related desynchronization.


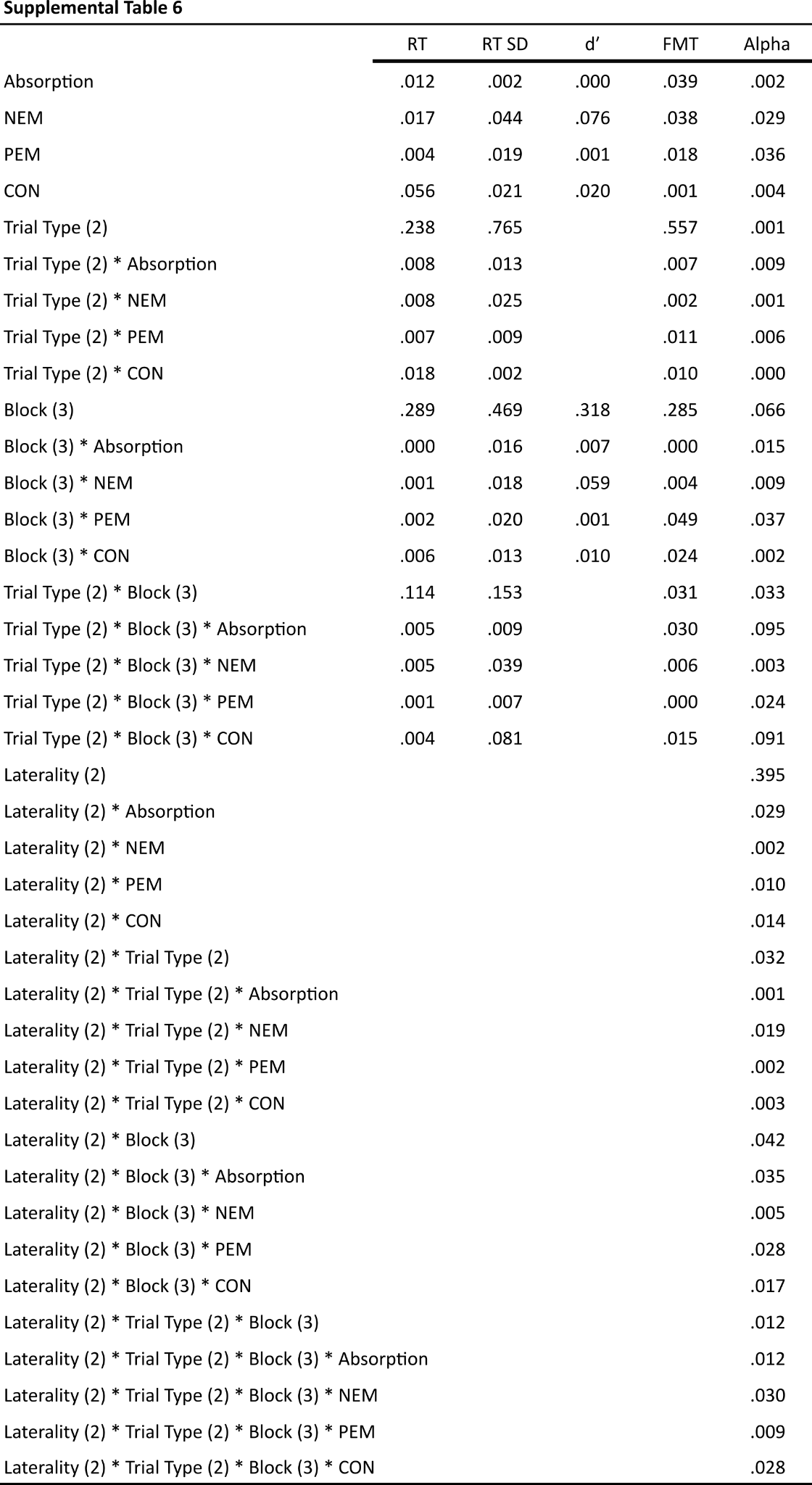


**Supplemental Table 6.** Effect size η_p_^2^ for general linear models, which were run without a random effect of subject (unlike the models reported in the main manuscript). Each separate model is represented with its own column.


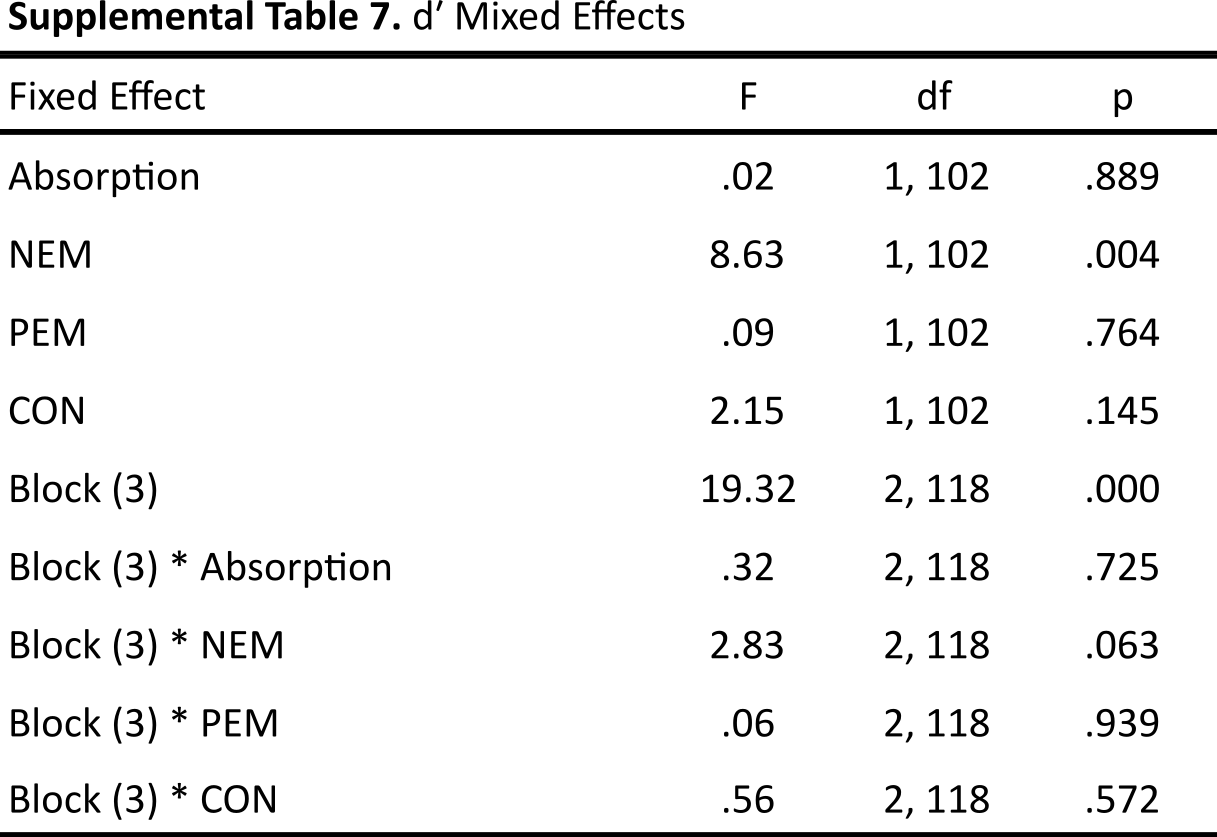


**Supplemental Table 7.** Full MIXED model statistics for analysis of d′ using a random effect of subject. Note that all MPQ scores and d′ data were z-scored before being entered into the model.

**
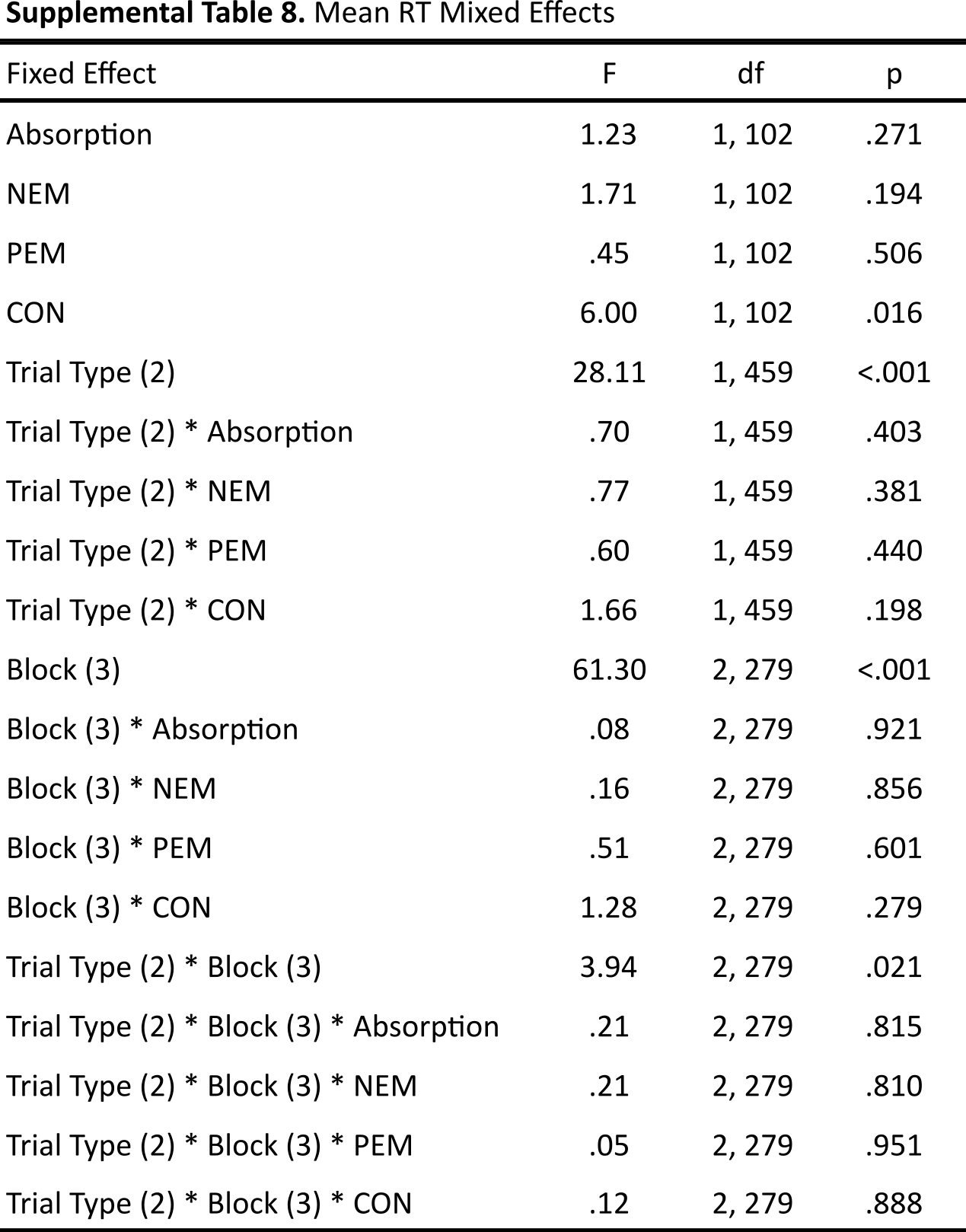
**

**Supplemental Table 8.** Full MIXED model statistics for analysis of mean RT using a random effect of subject. Note that all MPQ scores and RT data were z-scored before being entered into the model.


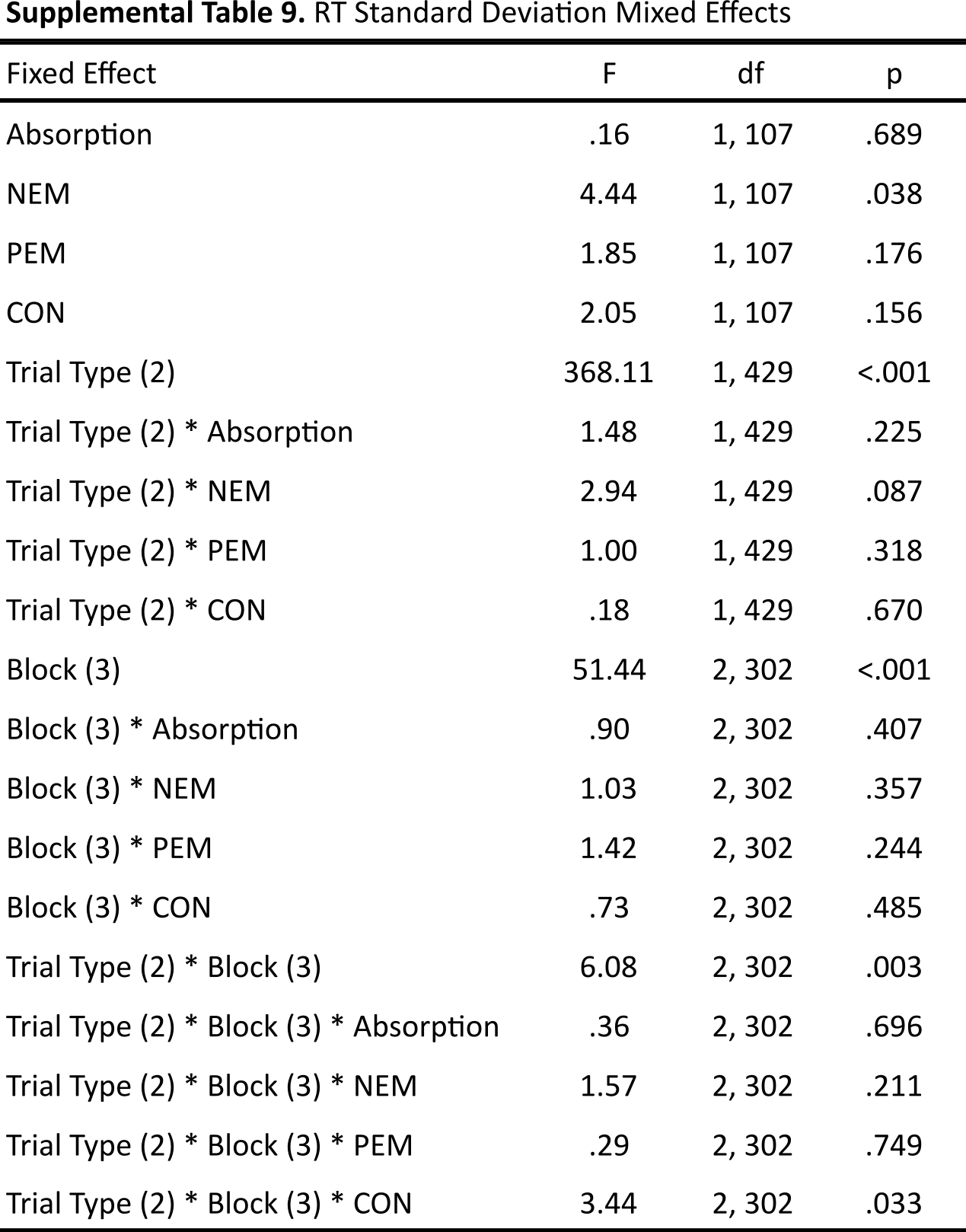


**Supplemental Table 9.** Full MIXED model statistics for analysis of RT SD using a random effect of subject. Note that all MPQ scores and RT SD data were z-scored before being entered into the model.


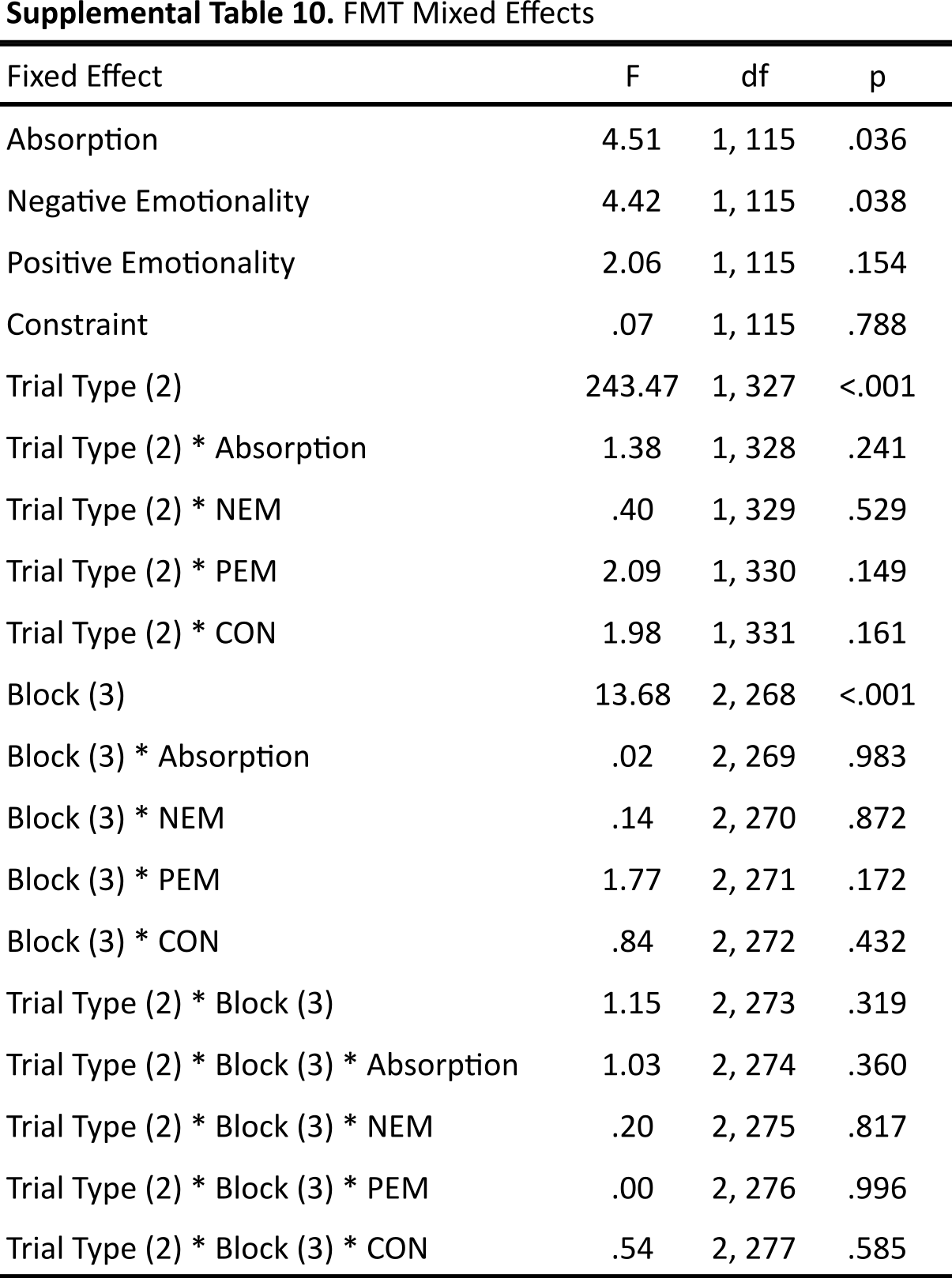
**Supplemental Table 10.** Full MIXED model statistics for analysis of FMT using a random effect of subject. Note that all MPQ scores and FMT data were z-scored before being entered into the model, and only correct trials were included.


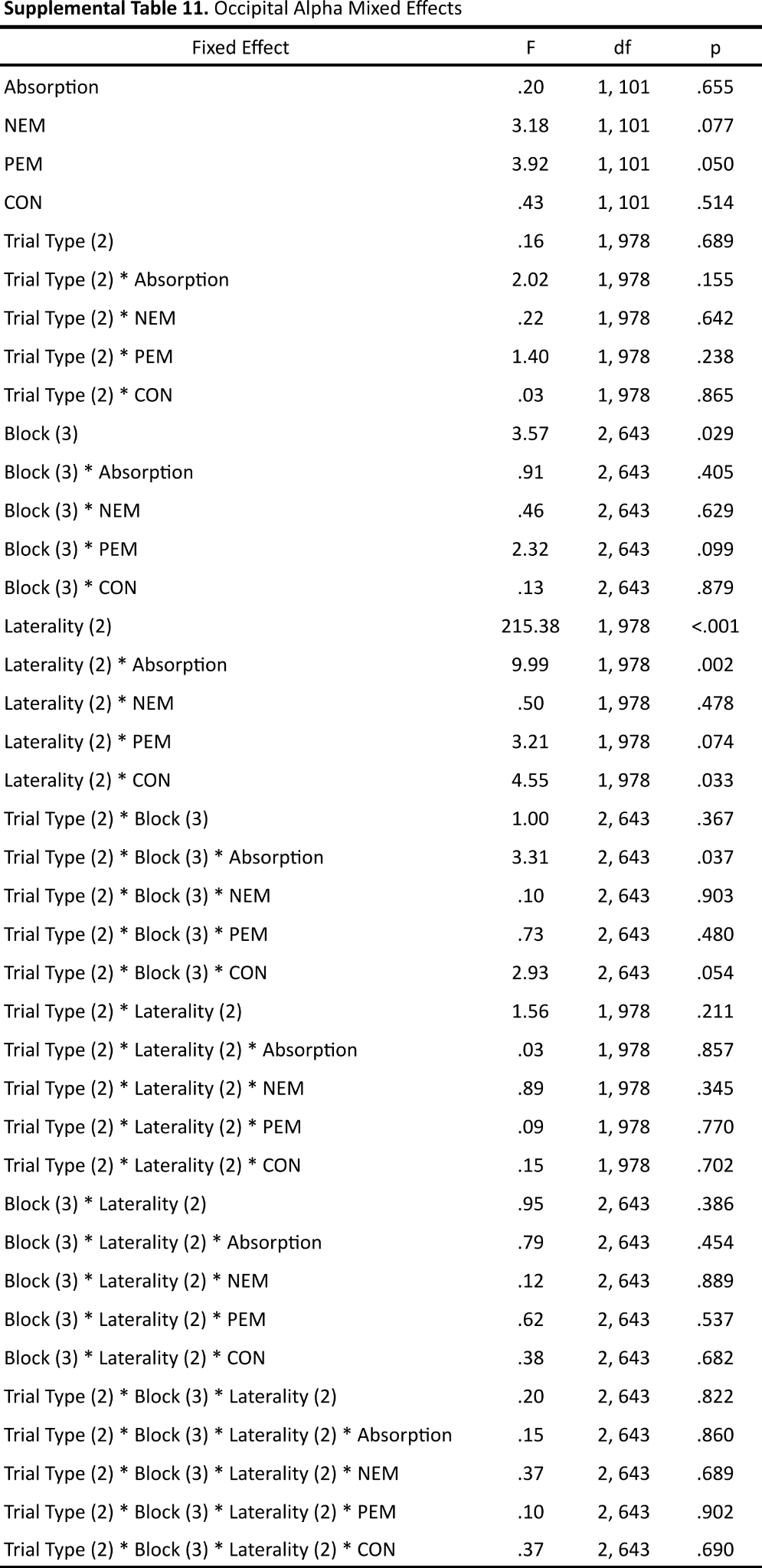


**Supplemental Table 11.** Full MIXED model statistics for analysis of occipital alpha using a random effect of subject. Note that all MPQ scores and alpha data were z-scored before being entered into the model, and only correct trials were included.

**Figure S3.** Correlation matrix of FMT and all other study variables.


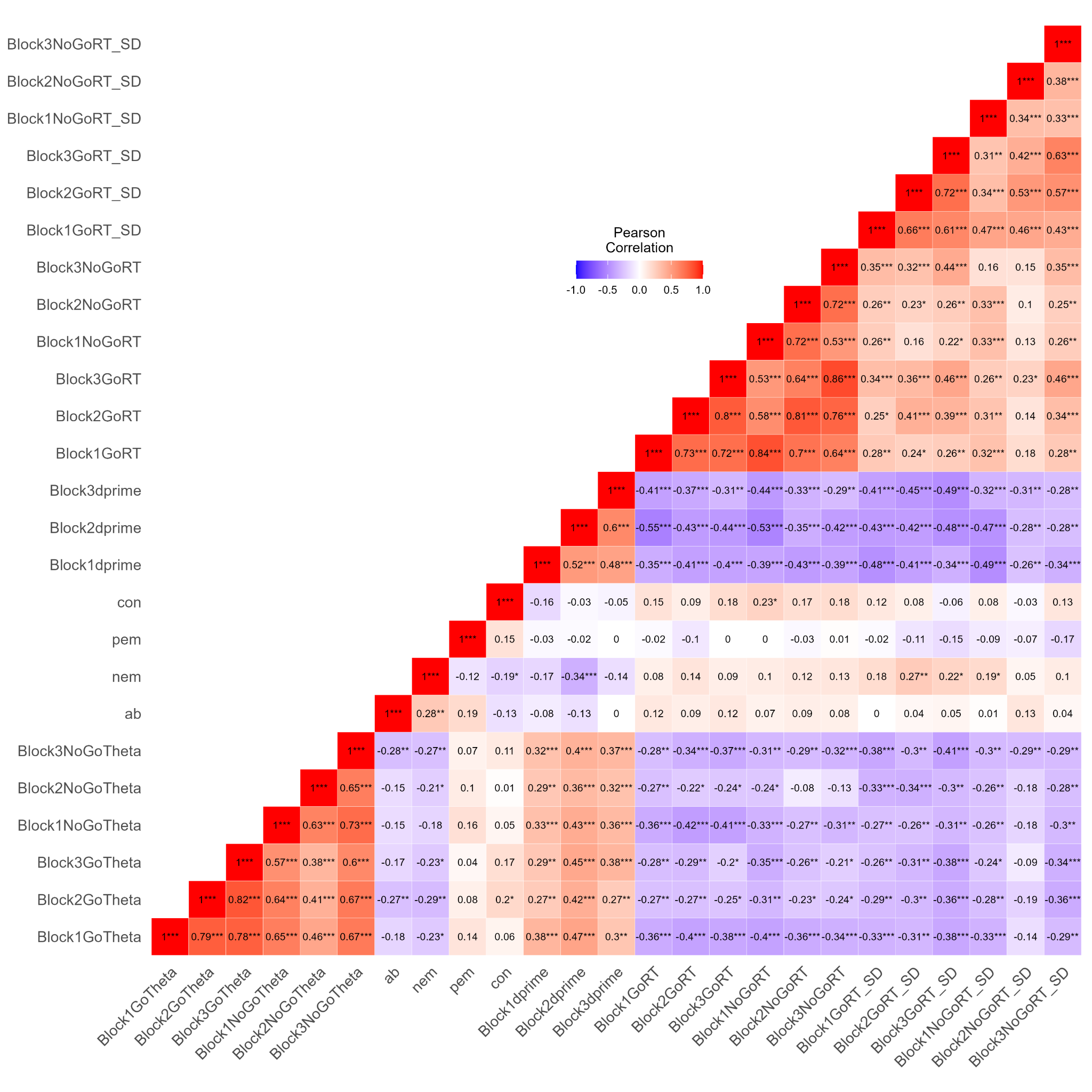


**Note.** * = <.05, ** = <.01, *** = <.001. RT = (Mean) Response Time, RT_SD = RT Standard Deviation, ab = Absorption, nem = Negative Emotionality, pem = Positive Emotionality, con = Constraint.

**Figure S4.** Correlation matrix of alpha and all other study variables.


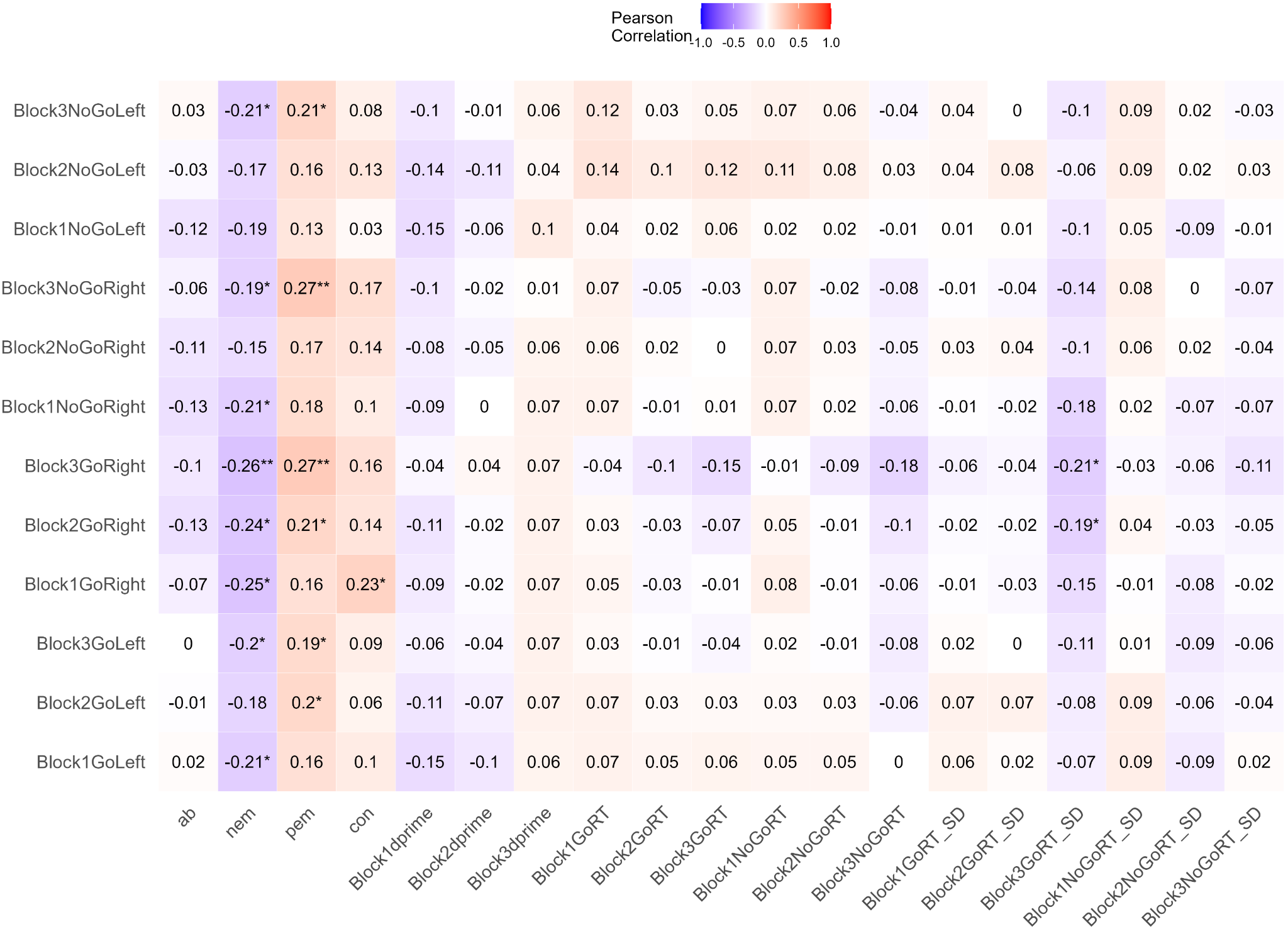


**Note.** * = <.05, ** = <.01, *** = <.001. Correlations between MPQ and behavioral variables not shown (see Figure S3).


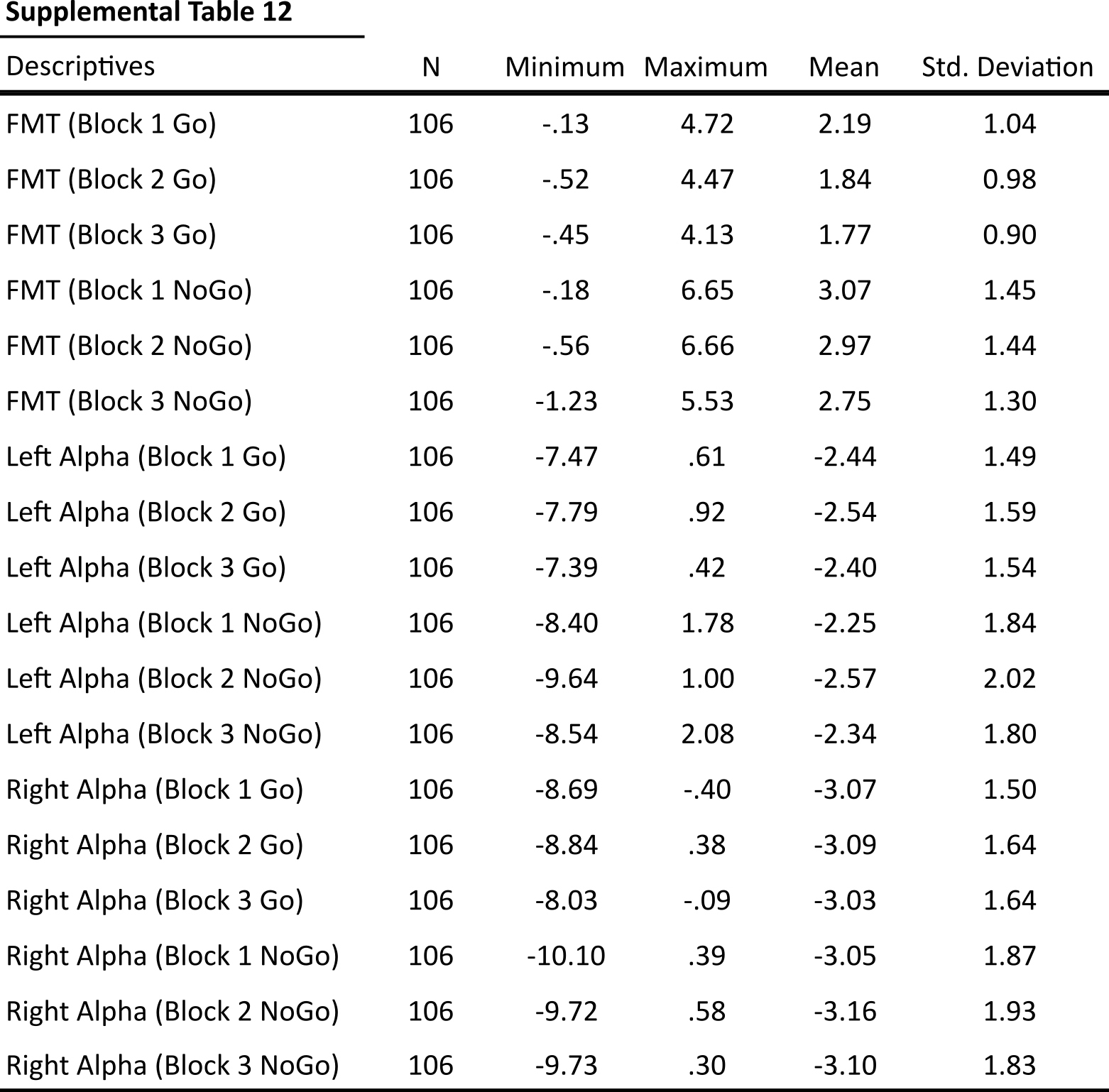


**Supplemental Table 12.** Descriptive statistics for all EEG variables from correct trials only.
